# Understanding the Dynamics of Blast Resistance in Rice-*Magnaporthe oryzae* Interactions

**DOI:** 10.3390/jof8060584

**Published:** 2022-05-30

**Authors:** Basavantraya N. Devanna, Priyanka Jain, Amolkumar U. Solanke, Alok Das, Shallu Thakur, Pankaj K. Singh, Mandeep Kumari, Himanshu Dubey, Rajdeep Jaswal, Deepak Pawar, Ritu Kapoor, Jyoti Singh, Kirti Arora, Banita Kumari Saklani, Chandrappa AnilKumar, Sheshu Madhav Maganti, Humira Sonah, Rupesh Deshmukh, Rajeev Rathour, Tilak Raj Sharma

**Affiliations:** 1 ICAR-National Rice Research Institute, Cuttack 753006, India; devnova2460@gmail.com (B.N.D.); anilcgpb@gmail.com (C.A.); 2 ICAR-National Institute for Plant Biotechnology, Pusa Campus, New Delhi 110012, India; priybioinfo@gmail.com (P.J.); amolsgene@gmail.com (A.U.S.); pkmolbio@gmail.com (P.K.S.); mandeep.dharwal@gmail.com (M.K.); hemu.bt@gmail.com (H.D.); jyoti.singh21@gmail.com (J.S.); chugh.kirti29@gmail.com (K.A.); banitakumari83@gmail.com (B.K.S.); 3 ICAR-Indian Institute of Pulses Research, Kanpur 208024, India; alokbio@gmail.com (A.D.); shallu.thakur85@gmail.com (S.T.); 4 National Agri-Food Biotechnology Institute, Mohali 140306, India; rajdeepjaswal52@gmail.com (R.J.); ritukapoor1985@gmail.com (R.K.); biohuma@gmail.com (H.S.); rupesh0deshmukh@gmail.com (R.D.); 5 ICAR-Directorate of Weed Research, Maharajpur, Jabalpur 482004, India; pawardv1@gmail.com; 6 ICAR-Indian Institute of Rice Research, Hyderabad 500030, India; sheshu24@gmail.com; 7 Department of Agricultural Biotechnology, CSK Himachal Pradesh Agricultural University, Palampur 176062, India; rathour72@gmail.com; 8 Indian Council of Agricultural Research, Division of Crop Science, Krishi Bhavan, New Delhi 110001, India

**Keywords:** rice, *Magnaporthe*, resistance, *R*-genes, QTLs, resistance-breeding, CRISPR/Cas

## Abstract

Rice is a global food grain crop for more than one-third of the human population and a source for food and nutritional security. Rice production is subjected to various stresses; blast disease caused by *Magnaporthe oryzae* is one of the major biotic stresses that has the potential to destroy total crop under severe conditions. In the present review, we discuss the importance of rice and blast disease in the present and future global context, genomics and molecular biology of blast pathogen and rice, and the molecular interplay between rice–*M. oryzae* interaction governed by different gene interaction models. We also elaborated in detail on *M. oryzae* effector and *Avr* genes, and the role of noncoding RNAs in disease development. Further, rice blast resistance QTLs; resistance (*R*) genes; and alleles identified, cloned, and characterized are discussed. We also discuss the utilization of QTLs and *R* genes for blast resistance through conventional breeding and transgenic approaches. Finally, we review the demonstrated examples and potential applications of the latest genome-editing tools in understanding and managing blast disease in rice.

## 1. Introduction

Rice, being the major staple food and one of the main sources of income and employment, is an important crop all over the world. Almost 90% of the global production and consumption of rice is reported from Asia, where a considerably large part of the world’s population resides (www.fao.org; accessed on 20 January 2022).

Since 2000, global rice consumption has exceeded its production and the annual shortage of rice is estimated to increase from 400,000 tons in 2016 to 800,000 tons by 2030 [1,2]. As the global population is expected to reach 9.77 billion by 2050, the rice production needs to be doubled from the present levels to ensure both global food and nutritional security. The total rice production can be enhanced by either increasing area of cultivation, productivity, or by avoiding the yield losses. However, the global analysis shows no scope for expanding the area due to the unavailability of arable land [3]. Therefore, avoiding the losses due to adverse environmental factors and post-harvest losses is the only feasible way to enhance the productivity and overall production.

Rice crop is challenged by a number of biotic and abiotic stresses in the various rice-growing regions of the world. Among these, blast disease caused by *M. oryzae* is considered as the most serious disease of rice [4]. The average losses due to this disease range from 10 to 30%, with up to 100% loss having been reported under severe conditions. In this article, we presented a critical analysis of the literature on the recent developments in understanding the rice–*M. oryzae* interaction, and understanding the complex mechanism of host resistance. Further, we elaborated on different approaches for the development of blast-resistant rice lines to counteract the imminent threat posed by the emergence of new *M. oryzae* races.

## 2. Global and National Significance of Rice Blast in Present and Future Context

Among the biotic stresses, rice blast is the most serious constraint that restricts the global rice production [4,5]. The major blast epidemics covering vast areas occur on a regular basis, resulting in 10–30% crop losses annually which represents a yield loss of about 157 million tons worldwide. The disease was reported for the first time in China in 1637 and was recognized as rice fever disease. Further, it was described as imochibyo in Japan in 1704 and as brusone in Italy in 1828. Now this disease covers almost 85 nations, posing a major threat to food security and farmers’ revenue in the regions of South Asia and Africa [6,7]. *M. oryzae* causes blast in more than 50 grass species [8], and among agriculturally important crop species, it infects rice, wheat, rye, barley, pearl millet, and finger millet.

Several blast epidemics have been recorded in rice. In the epidemic year 1953, an estimated loss of about 800,000 tons of rice was recorded in Japan. In India, blast disease was first reported in 1913, and in the year 1919 an epiphytotic was reported from the erstwhile Madras state [4]. Further, seven epidemics of rice blast were recorded in Himachal Pradesh, Andhra Pradesh, Tamil Nadu, and Haryana between 1980 and 1987 [4]. Even though huge yield losses have resulted due to rice blast epidemics, proper information on yield loss data during the last 30 years is not available for India. Frequent outbreaks have been recorded in hilly areas of Uttaranchal, Himachal Pradesh, and Jammu and Kashmir, where about 65% yield is lost to blast disease due to the prevailing blast-favorable condition during the kharif season [9]. Further, eastern India experiences frequent occurrences of the blast due to the favorable climatic conditions for the growth and development of the pathogen. About 564,000 tons of rice is lost yearly due to blast in eastern India alone, nearly 50% of which is lost under the upland ecosystem [9]. Blast disease incidence has also been reported in the rice-growing areas of peninsular and plain regions of India.

## 3. Molecular Interplay between Rice and *M. oryzae*

### 3.1. The Pathogen: M. oryzae

**Figure 1:**
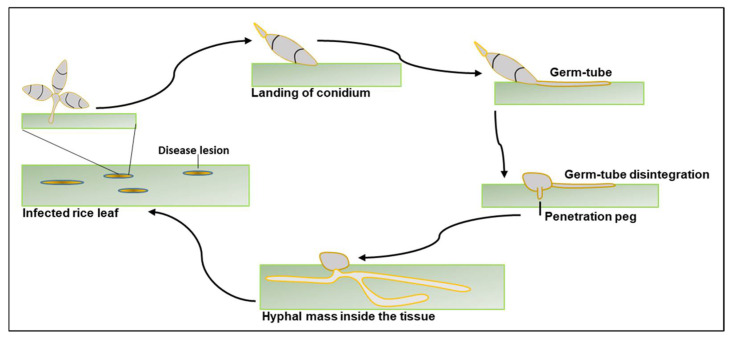
Schematic representation of the life cycle of the *M. oryzae* in rice.

*Magnaporthe oryzae*, the causal agent of blast disease in rice, is a hemibiotrophic pathogen belonging to the Magnaporthaceae family. This fungus attacks rice plants at all developmental stages and infects leaves, stems, nodes, panicles, and roots. The process of infection begins with the landing and attachment of a conidium to the rice leaf cuticle. An adhesive from the germinating conidium helps to stick to the cuticle. The conidium under favourable conditions germinates to produce germ-tube, which further differentiates into appressorium. The appressorium has a differentiated cell wall and a distinct melanin layer between its cell wall and the cell membrane. This layer helps in the generation of turgor pressure, which is later translated into mechanical force through the penetration peg and helps in penetration through the leaf cuticle. Once inside the cell, hyphae multiply rapidly, leading to disease development and visible blast symptoms. A graphical representation of the infection cycle of *M. oryzae* in rice is given in Figure 1. For detailed information about the infection process of *M. oryzae* in rice, readers can refer to the article by Wilson and Talbot [10].

#### 3.1.1. *Magnaporthe oryzae* Genomics

To develop durable and effective blast resistance in rice crop against *M. oryzae*, a comprehensive understanding of the molecular mechanism underlying the blast pathogenesis and host resistance in rice required. The developments in the field of genomics, associated with various molecular biology techniques, are enabling researchers to dissect out the molecular mechanism of rice–*M. oryzae* interaction.

Among the phytopathogenic fungi, *M. oryzae* is the first one to have its genome sequenced [11]. This fungus has emerged as a model system for studying the host–pathogen interactions and understanding the pathogenicity mechanisms of plant pathogens. *M. oryzae* displays an exceptional genomic plasticity due to frequent occurrences of repeat elements in its genome. Such genome instability leads to a rapid evolution of new races in the population. Genome resequencing of *M. oryzae* provides an opportunity to investigate and understand the host–pathogen interaction processes of a particular strain at the molecular level for effectively managing the rice blast disease [12]. Consequently, genomes of more than 74 strains of *M. oryzae* have been sequenced (Table 1). On average, the genome size of *M. oryzae* is 40.12 Mb and contains 12,684 genes (Table 1). However, the estimated genome size with a gap was reported to be 40.3 Mb [11]. Repeat elements distribution throughout its genome is the main factor that determines the genome plasticity and several studies have reported the influence of repeat elements in the genomic features [12,13]. Many avirulence and effector molecules have been characterized in this pathogen and were found to have association with the repeat element for their functional activities [12]. The repeat elements are known to play a vital role in *M. oryzae* genome variation and genome evolution, and largely impact the virulence spectrum at the individual strain level [13,14,15]. Interestingly, every sequenced *M. oryzae* strain had isolate-specific genomic regions as well as genes. Such isolate-specific genes and genomic regions determine racial evolution, environmental adaptation, chromosomal variability, variation in repeat element distribution, and host range specificity during the course of evolution [14,16]. It is also hypothesized that the isolate-specific genes might be an event of the gene gained or lost during the evolution process [13]. The resequencing of different *M. oryzae* genomes assists in constructing a pan-genome that describes a consensus genome sequence derived from multiple or individual genomes of different strains, species, or genera, and could be utilized as a complete reference sequence. Pan-genome has many prospective applications and helps to analyze multi-omics data. It could be used for genome-wide association study, metagenomics, population genetics, phylogenomics, etc. Pan-genome also provides information about the presence and absence of variation, core genes, dispensable genes, etc. Using the pan-genome approach in *M. oryzae*, Singh et al. [12] identified a retro-transposable element that displayed a significant difference in copy number and distribution between virulent and avirulent strains. The knowledge gathered through different genomics resources by applying bioinformatics approaches such as comparative genome analysis, pan-genome analysis, and metagenomics analysis can shed light on the mechanisms of frequent emergence of new races of *M. oryzae,* and advances our understanding about the host–pathogen interactions for the effective management of blast disease.

#### 3.1.2. Pathogenicity Related Factors of *M. oryzae*

i. Analysis of *Avr* and effector genes. Pathogen-associated molecular patterns (PAMPs) are recognized by host cell surface-localized pattern-recognition receptors (PRRs) to activate plant immunity. PAMP-triggered immunity (PTI) constitutes the first layer of plant immunity that restricts pathogen proliferation. To establish a successful infection, the *M. oryzae* has to overcome the PTI. Therefore, a successful isolate switches to the deployment of effector proteins, leading to a response known as effector-triggered susceptibility (ETS) [26]. In ETS, the effector plays with host defense regulators, such as the Secreted LysM protein 1 (Slp1) with two LysM domains; acts as a competitive inhibitor for the binding of chitin with host chitin elicitor-binding protein (CEBiP); and thereby subverts the PTI [16]. A recent study demonstrated a similar mechanism, where chitinase 1 (MoChia1) competitively inhibits the binding of host tetratricopeptide repeat protein (OsTPR1) with PAMP, chitin, and compromises PTI [27]. More recently, effectors such as MoHTR1 and MoHTR2 were found to be directly targeted into the rice nucleus to undermine PTI [28]. Till-date, 26 Avr/effector genes have been mapped in *M. oryzae*, and 14 of them, including two unmapped Avrs, *MoHTR1* and *MoHTR2*, have been cloned and characterized (Table 2).

The first discovered Pwl effectors (Pwl1–Pwl4) belong to a small, glycine-rich rapidly evolving effector family that provides avirulence on weeping lovegrass and finger millet, but has no effect on rice. Except for cell death-induction/suppression or interacting with resistance proteins features, the identification of candidate effector proteins is a difficult task due to their unique sequence features. Recent structural studies have shown that despite sequence divergence, several effector proteins can share structural similarities. An NMR-based study on diverse ascomyceteous effectors from *M. oryzae* and *Pyrenophora tritici*-repentis revealed these proteins to possess conserved 6 β-sandwich structures stabilized by conserved cysteines. A detailed study showed that previously characterized effectors such as Avr Piz-t and ToxB also possess those folds, forming a conserved MAX effector family (*Magnaporthe* Avrs and ToxB like). Additionally, 5–10% of the effectors expressed in the biotrophic phase of *M. oryzae* possess MAX, and more than 50% of cloned effectors also belong to the MAX effector class. The computation prediction of the effector proved to be a useful method to highlight potential candidates with conserved folds.

Among the 26 reported *Avr*-genes, 15 were mapped near the chromosome ends, and 5 of the cloned *Avr* genes were flanked by transposons. These transposons are active companions of the *Avr* genes and play a role in the loss and gain of these genes. The molecular interaction studies of the reported seven R-Avr pairs showed that five of them, namely, Pi-ta/AVR-Pita, Pik/AVR-Pik, Pia/AVR-Pia, Pi-CO39/AVR1-CO39, and Pi54/AVR-Pi54 interact directly, whereas Piz-t/AvrPiz-t and Pii/AVR-Pii have indirect interaction. Besides the one-to-one interactions, two other types of interactions were also reported. One, where two R proteins (homologs) interact with one Avr protein, such as in Pik-1 and Pik-2 with AVR-Pik [43], and a similar mechanism is predicted in the case of recently cloned Avr *MoHTR1* and *MoHTR2*, which are predicted to interact with the same target protein [28]. In another interaction, two different Avr proteins could be recognized by a single R protein complex, such as two NLR proteins, RGA4 and RGA5 hetero-dimers interacting with AVR-Pia or AVR1-CO39 [44,45].

ii. Small non-coding RNAs in pathogenesis. Eukaryotic organisms produce small RNAs (sRNAs) that include microRNAs (miRNAs) and short interfering RNAs (siRNAs) of approximately 18–25 base pairs. These sRNAs modulate the diverse cellular activities through a process known as RNA interference (RNAi) [46]. These RNAs also take part in the mechanisms to subvert the host immune system during infection [47]. Most of the sRNAs produced by the organisms’ function endogenously, however, there are reports suggesting that these sRNAs can travel beyond the organismal boundaries and regulate the genes in interacting organisms through a mechanism commonly known as “trans-kingdom RNAi” [46,48]. This bidirectional movement of sRNAs has been reported between animal/plant hosts and microorganisms interacting with them [48,49,50,51,52,53].

*Magnaporthe oryzae* possess functional RNAi pathway genes such as Dicer, Argonaute, and RNA-dependent RNA polymerase. These genes are required for the biogenesis of sRNAs that are involved in the regulation of fungal growth, virulence, and stress tolerance [54,55]. Nunes et al. [56] characterized the sRNA repertoire in this pathogen by using the next-generation sequencing approach. They reported tissue-specific enrichment of sRNAs from mycelia and appressoria-specific small RNA libraries. Raman et al. [54] analyzed the expression sRNAs in *M. oryzae* by subjecting it to different *in-vitro* stresses and observed two distinct peaks of sRNAs of 24 nt and 26 nt during mycelial and in-planta growth, respectively [55]. The majority of sRNAs produced by this pathogen were aligned to intergenic (54%) and repeat regions (41%) and only 4% of the total sRNAs matched to protein-coding genes. Although the numbers of sRNAs mapped to intergenic and repeat regions was high, the proportion of uniquely mapped sRNAs was higher in protein-coding regions [54]. The sRNA expression profiles of the pathogen indicated differential preference for 5′ nucleotide; the most abundant nucleotide at the start of sRNAs was Uracil followed by Guanine in mycelial and Adenine in in-planta libraries. By employing a knockout mutant of Argonaute (Δ*moago3*) and RNA-dependent RNA polymerase (Δ*mordrp1*), Raman et al. [55] demonstrated that sRNAs produced by the pathogen are involved in the regulation of pathogenesis-related genes such as *MGG_01662* (4-aminobutyrate aminotransferase), *MGG_02329* (isotrichodermin C-15 hydroxylase), and *MGG_02378* (glutamate decarboxylase). *M. oryzae* mutants lacking *moago3* and *mordrp1* failed to infect barley during wound assays, and both mutants showed reduced production conidia. The deletion of a single *Dicer* (Δ*modcl*2) or double knockout for *Dicer* genes (Δ*modcl1*+Δ*modcl*2) upregulated many genes involved in pathogenicity such as *MGG_10932* coding for C2H2 zinc-finger transcription factor involved in the formation of appressorium*; MGG_14068*, encoding a putative FAD oxidoreductase; *MGG_02065* encoding kinesin light chain; and MGG_10027 encoding a calcium-transporting ATPase 1.

The above studies clearly demonstrated that the RNAi pathway genes and sRNAs play an important role in the regulation of the pathogenicity during rice–*M. oryzae* interaction. Besides several proteinaceous and non-proteinaceous effector molecules [57], many fungal pathogens have developed novel virulence mechanisms and deliver sRNAs as effector molecules to overcome immunity during interaction with the host plant [52,58,59]. In *M. oryzae,* the comparative expression profiling of sRNAs during vegetative phase and rice infection revealed a significant upregulation of 366 *M. oryzae* sRNAs during infection. Out of these differentially expressed sRNAs, 14 were identified as potential candidates, which may act as effectors during rice–*M. oryzae* interaction [60]. In another study, Zhao et al. [46] computationally created a regulatory network of *M. oryzae* sRNAs during rice–*M. oryzae* interaction by utilizing the available transcriptomics and proteomics resources. These researchers identified 22 sRNAs and 77 secretory proteins of *M. oryzae* that may participate in the infection process. Though both the above studies predicted *M. oryzae* sRNAs as possible mediators of rice–*M. oryzae* interaction, the accuracy of *in-silico* findings needs to be further verified through extensive laboratory studies.

#### 3.1.3. Editing Pathogenicity Genes in *M. oryzae*

CRISPR-Cas systems have been extensively used in editing the genomes of diverse organisms for a wide range of applications [61,62]. For a wide range of filamentous fungus, including various plant pathogens, CRISPR/Cas-based genome-editing strategies have already been demonstrated [61,62]. They are usually based on Cas9 and gRNA transgenic expression. Cas9 and gRNA coding plasmid can be transformed to fungal cells; however, sometimes they might prove cytotoxic. To avoid cytotoxicity, Cas9 and gRNA can also be complexed into a functional ribonucleoprotein (RNP) *in-vitro* and then introduced through transformation, as established in *M. oryzae* [62,63]. gRNA molecules may be synthesized in vitro and co-transformed alongside Cas9-encoding cassettes. In microalgae, in vitro synthesized and characterized gRNAs were supplied as RNPs along with Cas proteins [64,65,66,67]. In the rice blast fungus *M. oryzae*, concurrent double editing was accomplished using this approach [63]. Similarly, Cas12a RNP was successfully used for *M. oryzae* genome editing in a recent report [68]. However, there are no reports on CRISPR/Cas-based transformation of *M. oryzae* for changing pathogenicity or creating novel variants of the pathogen.

#### 3.1.4. *Magnaporthe* Host-Shifting

*Magnaporthe oryzae* is known to infect many crops including rice, wheat, several millets, and grasses among the others. The rice blast pathogen (*M. oryzae* pathotype *Oryza*: MoO) is ranked top in the list of 10 most destructive fungal plant pathogens [69], whereas wheat blast (WB) pathogen *M. oryzae* pathotype *Triticum* (MoT) was a lesser known disease with its confinement to parts of Africa. The recent wheat blast epidemics in Bangladesh and Zambia [70,71], however, put the MoT on the global map for having a serious consequence on the world wheat production. Besides other factors, the prevailing weather conditions during the wheat-growing season seem to have played a major role in wheat blast epidemics in Bangladesh [72,73]. Though initially it was assumed that WB is an event of host-shift of MoO from rice to wheat, recent molecular studies have confirmed that MoT from Bangladesh and South America have highly similar genetic content [74], and they are also distinct from other *Magnaporthe* pathotypes [75,76,77,78]. Interestingly, among the *Magnaporthe* pathotypes, only *Triticum* (MoT) infects other hosts than its main host, wheat [79,80]. These findings highlight the potential host-shift/jumping of *Magnaporthe* pathotypes and the associated threat to the crop production, specifically to cereals and millets.

### 3.2. The Host: Rice

#### 3.2.1. Genomes Sequenced

The genome *Oryza* is the smallest cereal crop genome with an estimated size of 400–430 Mb [81]. *Oryza sativa japonica* cultivar *Nipponbare* was the first rice genome sequenced [81]. However, the *O. sativa indica* rice, which further has *indica* and *aus* subpopulations, is the most widely planted rice globally [82]. Several draft genomes of *indica* rice have been assembled [83]. A highly contiguous and near-complete *indica* rice genome reported is for the cultivar Shuhui 498 (R498) [84]. The developments in the field of next-generation sequencing (NGS) technologies have revolutionized the field of genome sequencing in plants [85]. In a major effort, a core collection of 3,000 rice accessions from 89 countries were sequenced with an average sequencing depth of 14 X, average genome coverage of 94.0%, and average mapping rates of 92.5% [86]. After aligning these genomes to the reference genome, i.e., Nipponbare, a total of 18.9 million single nucleotide polymorphisms (SNPs) were discovered. SNP data were used for the phylogenetic analyses to differentiate *O. sativa* gene pool into five varietal groups (*indica*, aus/boro, basmati/sadri, tropical *japonica*, and temperate *japonica)*. Besides *O. sativa*, genomes of different wild species of rice have been sequenced. The list of wild rices with their genomes sequenced include, *O. longistaminata* (2014; ID: 11285); *O. glaberrima,* an African wild rice (2010; ID:458); *O. minuta*, a perennial wild rice from southeast Asia (2014; ID: 10965); *O. meridionalis, a* wild rice from Australia (2012; ID: 11319); *O. coarctata*, a wild rice from Bangladesh (2019; ID: 11313); *O. australiensis*, an Australian wild rice variety (2021; ID: 10966); *O. officinalis*, a tropical and sub-tropical wild rice (2014; ID: 10964); *O. punctata*, an African wild rice (2014; ID: 10963); *O. nivara*, a wild rice from India (2014; ID: 2841); *O. rufipogon*, a wild rice species from tropical and subtropical regions of Australia and Asia (2014; ID: 457),;*O. meyeriana var*. granulata, a wild rice from Thailand (2012; ID: 11287); and *O. glumipatula* (2013; ID: 11318) [72].

With the availability of a large number of genome sequences within rice, such as in 3K database [87], efforts are being made to study the rice genome at the pan-genome level. Zhao et al. [88] constructed a pan-genome dataset of the *O. sativa–O. rufipogon* species complex through sequencing and de novo assembly in 66 diverse accessions. Most of the stress response-related genes, including those coding for NBS-LRR proteins, were detected only in a subset of accessions, thus suggesting the existence of a diverse repertoire of biotic stress-resistance genes in the species studied. Therefore, pan-genome studies in rice hold a greater potential for the identification of new blast resistance genes.

#### 3.2.2. Resistance Genes as Solo Protectors

Resistance (*R*) genes are an integral part of the plant defensome complex, and *R* and defense response (*DR*) genes contribute to broad-spectrum blast resistance in rice [89,90]. The immune responses governed by most of these *R* genes have intertwined networks and they mostly regulate downstream general defense pathways [91]. To date, more than 100 blast *R* genes have been mapped in rice and 38 of them have been cloned and characterized [Table 3 and Table 4]. As a result of comprehensive genetic studies in rice, vis-à-vis its pathogen, *M. oryzae*, the rice–*M. oryzae* interaction has emerged as a premier model system for understanding the plant–fungal pathogen interactions [4,92]. The narrow race-specificity of *R*-genes and the ability of the pathogen to quickly evolve new races compatible with resistance genes are major hurdles in achieving long-lasting protection against the blast disease [93]. The identification and utilization of *R* genes that confer broad-spectrum resistance against a large number of pathogen races is the most effective approach to manage the disease [91]. Interestingly, among these 38 blast *R* genes, eight, namely *Pi9* [94], *Pi54* [95], pi*21* [96], *Pi50* [97], *Pi7* [98], *Pi57* [99], *Pigm* [100], and *Ptr* [101], are reported to provide broad-spectrum blast resistance, and only three, *Pi5-1*, *Pi63,* and *Pb1* are pathogen-inducible, and the rest are expressed constitutively. Recently, Wang et al. [102] performed genome-wide analysis of NBS-LRR genes in a broad-spectrum resistant rice genotype Tetep, and functionally validated the role of 90 NBS-LRR genes in blast resistance. Since these genes have not been assigned designations as per the standard conventions for naming blast resistance genes in rice, these genes have not been included in the list of cloned *R* genes provided in Table 4.

The *R* genes generally act solo to induce the immune response in rice. However, the downstream pathways leading to resistance response encompass one or more DR proteins [91]. It is found that at least some of the reported *R* and *DR* gene combinations show positive association for conferring blast resistance in rice e.g., *Pid2* with *OsPUB15*, *Pik-H4* with *OsBIHD1,* and *Pita* with lesion mimic (sl) gene [103,104,105]. Besides *R* genes, five DR genes *bsr-d1*, *bsr-k1*, *OsBBI1*, *spl11,* and *spl33* are reported to confer broad-spectrum blast resistance [69]. Among the DR genes identified for broad-spectrum blast resistance in rice [91], rice lesion mimic mutants (*lmm*) genes form a major section of these genes. These LMM mutants, through programmed cell death (PCD), mimic the blast symptoms and the natural HR response by inducing the resistance response. There are more than 30 *lmm* genes reported in rice for blast resistance. However, most of these genes have associated yield penalties while imparting the immune response. Hence, *lmm* genes are of limited use for rice blast-resistance breeding [91,106].

**Table 3:** Information on mapped rice blast resistance genes.

Sl. No.	Gene ID	Chr. No.	Position (cM)	Source Cultivar	Reference
1	*Pit*	1	9.08–12.17	Tjahaja	[107]
2	*Pi27(t)*	1	24.29–27.90	IR64 (Indica)	[108]
3	*Pi24(t)*	1	20.97–22.22	Azuenca (Japonica)	[109]
4	*Pitp(t)*	1	100.54–108.43	Tetep	[110]
5	*Pi35(t)*	1	132.0–136.6	Hokkai 188 (Japonica)	[111]
6	*Pi37*	1	132.44–133.95	St. No. 1 (Japonica)	[112]
7	*Pish*	1	135.3-138.7	Shin2 (Japonica)	[113]
8	*Pid1(t)*	2	87.5–89.9	Digu	[114]
9	*Pig(t)*	2	137.38–140.54	Guangchangzhan (Indica)	[115]
10	*Pitq5*	2	150.5–157.9	Teqing	[116]
11	*Piy1(t)*	2	153.2–154.1	Yanxian No. 1	[117]
12	*Piy2(t)*	2	153.2–154.1	Yanxian No. 1	[117]
13	*Pib*	2	153.2–154.1	Tohoku IL9	[118]
14	*Pi25(t)*	2	137.44–150.90	IR64 (Indica)	[119]
15	*Pi14(t)*	2	1–26.90	Maowangu	[120]
16	*Pir-2-3(t)*	2	96.8–99.3	IR64 (Indica)	[121]
17	*Pitq2*	2		Teqing (Indica)	[122]
18	*Pirf2-1(t)*	2	109.6–112.2	*O. rufipogon* (W)	[121]
19	*Pi16(t)*	2	1–26.91	Aus373 (Indica)	[123]
20	*Pitq3*	3		Teqing (Indica)	[122]
21	*Pi68*	3	6.8-9.7	*O. glumaepatula* (W)	[124]
22	*pi21*	4	20.97–22.22	Owarihatamochi	[125]
23	*Pikur1*	4	98.44–134.23	Kuroka (Japonica)	[126]
24	*Pi39(t)*	4	107.4–108.2	Chubu 111 (Japonica)	[127]
25	*Pitq4*	4		Teqing (Indica)	[122]
26	*Pi(t)*	4	9.08–12.17	Tjahaja	[128]
27	*Pi26(t)*	5	35.00–46.70	Gumei 2 (Indica)	[119]
28	*Pi23(t)*	5	43.02–76.70	Sweon 365	[125]
29	*Pi10*	5	58.08–75.41	Tongil	[129]
30	*Pi22(t)*	6	19.5–24.09	Suweon365 (Japonica)	[125]
31	*Pi26(t)*	6	35.00–46.70	Azucena (Japonica)	[130]
32	*Pi27(t)*	6	22.22–2.97	IR64 (Indica)	[108]
33	*Pi40(t)*	6	65.09–70.12	*O. australiensis* (W)	[131]
34	*Piz*	6	40.6–42.07	Zenith (Japonica)	[132]
35	*Piz-t*	6	58.7	Toride 1	[107]
36	*Pi9*	6	41.5–41.55	*O. minuta* (W)	[133]
37	*Pi25(t)*	6	72.32–77.03	Gumei 2	[119]
38	*Pi8*	6	19.5–24.09	Kasalath (Indica)	[120]
39	*Pi3(t)*	6		Pai-kan-tao (Japonica)	[134]
40	*Pitq1*	6	92.6-98.2	Tequing (Indica)	[135]
41	*Pi13(t)*	6	56.8-60.5	Kasalath (Indica)	[136]
42	*Pii1*	6	88.8-90.6	Fujisaka 5 (Indica)	[120]
43	*Pid2*	6	68.63–68.65	Digu	[137]
44	*Pigm(t)*	6	41.47–41.68	Gumei 4	[138]
45	*Pi17(t)*	7	89.00–99.9	DJ 123	[120]
46	*Pi36*	8	11.48–11.53	Q61 (Indica)	[127]
47	*Pi33*	8	23.66–24.61	IR64 (Indica)	[134]
48	*Pizh*	8	17.48–84.04	Zhai-Ya-Quing8 (Indica)	[108]
49	*Pi11*	8		Zhai-Ya-Quing8 (Indica)	[128]
50	*Pi29(t)*	8	38.65–64.96	IR64 (Indica)	[108]
51	*Pii2(t)*	9	4.09–28.89	Azucena	[139]
52	*Pi5(t)*	9	31.3–33.0	RIL125, RIL249 and RIL260(Moroberekan)	[140]
53	*Pi3(t)*	9	31.3–33.1	Kan-Tao	[128]
54	*Pi15*	9	38.56–38.74	GA25 (Japonica)	[120]
55	*Pii*	9	9.16–113.72	Ishikari Shiroke (Japonica)	[141]
56	*Pi28(t)*	10	78.26–90.67	IR64 (Indica)	[108]
57	*Pia*	11	1.01–2.09	Aichi Asahi (Japonica)	[126]
58	*PiCO39(t)*	11	25.21–27.55	CO39 (Indica)	[142]
59	*Pilm2*	11	54.54–113.5	Lemont	[116]
60	*Pi30(t)*	11	1.76–26.31	IR64 (Indica)	[108]
61	*Pi7(t)*	11	71.4–84.3	RIL29 (Japonica)	[143]
62	*Pi34*	11	77.69–77.96	Chubu32 (Japonica)	[144]
63	*Pi38*	11	76.55–87.91	Tadukan (Indica)	[145]
64	*PBR*	11	80.5–120.3	St. No. 1	[146]
65	*Pb1*	11	85.7–91.4	Modan	[147]
66	*Pi44(t)*	11	91.4–117.9	RIL29 (Japonica)	[148]
67	*Pik-h* (*Pi54*)	11	99.0–99.05	Tetep	[95]
68	*Pi1*	11	105.99–113.49	LAC23 (Japonica)	[149]
69	*Pik-m*	11	109.25–110.13	Tsuyuake (Japonica)	[150]
70	*Pi18(t)*	11	107.18–113.50	Suweon365 (Japonica)	[132]
71	*Pik*	11	109.25–110.13	Kusabue (Indica)	[151]
72	*Pik-p*	11	109.25–110.14	HR22 (Indica)	[107]
73	*Pik-s*	11	109.25–110.15	Shin 2 (Japonica)	[152]
74	*Pik-g*	11	109.25–110.16	GA20 (Japonica)	[120]
75	*Pise1*	11	22.96–66.92	Sensho	[153]
76	*Pi f*	11	98.78–113.84	Chugoku 31-1 (St. No. 1)	[154]
77	*Mpiz*	11	16.29–66.92	Zenith (Japonica)	[155]
78	*Pikur2*	11	11.36–73.49	Kuroka (Japonica)	[125]
79	*Pish*	11	110.3–111.8	Nipponbare (Japonica)	[113]
80	*Pib2*	11	105.99–113.49	Lemont (Japonica)	[122]
81	*Pi44*	11	85.7–89.7	Moroberekan (Japonica)	[148]
82	*Pi47*	11		Xiangzi (Indica)	[116]
83	*Pise*	11	22.96–66.92	Sensho	[153]
84	*Piis1*	11	11.36–76.11	Imochi Shirazu (Japonica)	[153]
85	*Pi24(t)*	12	20.97–22.22	Azuenca (Japonica)	[156]
86	*Pi62(t)*	12	9.7–77	Tsuyuake (Japonica)	[157]
87	*Pitq6*	12	23.0–30.92	Tequing (Indica)	[116]
88	*Pi6(t)*	12	1–1.68	Apura (Indica)	[158]
89	*Pi12*	12	27.95–60.48	Moroberekan (Japonica)	[159]
90	*Pi21(t)*	12	20.94–22.22	Owarihatamochi (Japonica)	[125]
91	*Pi31(t)*	12	30.92–47.66	IR64 (Indica)	[108]
92	*Pi32(t)*	12	52.41–75.46	IR64 (Indica)	[108]
93	*Pi157*	12	49.5–62.2	Moroberekan (Japonica)	[123]
94	*Pita*	12	42.41–42.43	Tadukan (Indica)	[107]
95	*Pita-2*	12	40.31–52.84	Shimokita (Japonica)	[160]
96	*Pi19(t)*	12	35.30–53.67	Aichi Asahi (Japonica)	[161]
97	*Pi39(t)*	12	-	Chubu 111 (Japonica)	[127,162]
98	*Pi20(t)*	12	51.5–51.8	IR24 (Indica)	[163]
99	*Pi20*	12	49.6-50.4	IR24 (Indica)	[164]
100	*Pi42(t)*	12	58.9-56-7	DHR9 (Indica)	[151]
101	*Pi48*	12		Xiangzi 3150 (Indica)	[116]
102	*PiGD-3(t)*	12	55.8	Sanhuangzhan 2	[138]

**Figure 2:**
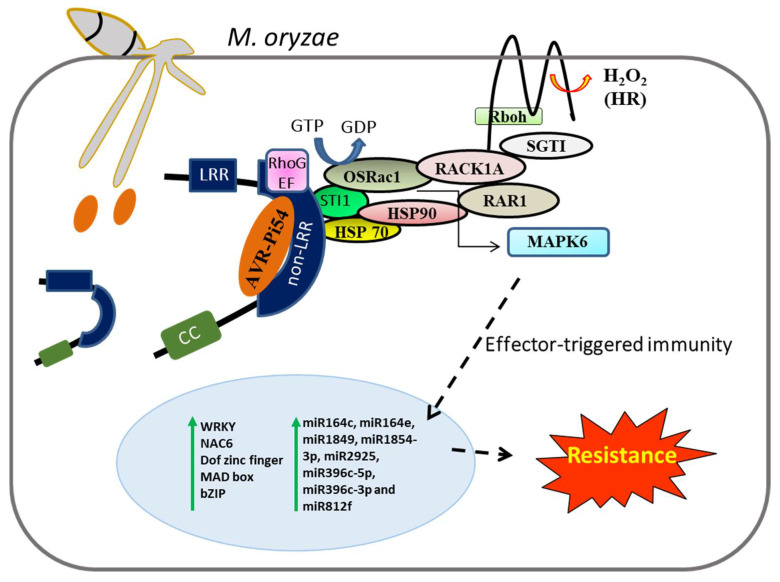
Schematic presentation of mechanisms of *Pi54* locus-mediated blast disease resistance. The Avr-Pi54 effector binds to Pi54 and its orthologs (blue) mainly at the non-LRR region, upstream to the LRR domain. The Pi54 loci perceive the *M. oryzae* signals through STI1, an anchor for defensome complex involving multiple proteins such as OsRac1 (Rac/Rop GTPase), RACK1A (Receptor of Activated C Kinase), RAR (Required for Mla12 Resistance), SGT1 (Suppressor of the G2 allele of skp1), MAPK6 (a rice Mitogen-Activated Protein Kinase), and Rboh (NADPH oxidases). The MAPK6-mediated downstream signaling pathways might induce various *DR* genes. Besides, a set of miRNAs induced by the Pi54 loci are playing a role disease-resistance response. This figure is partially adapted and modified with the latest information [89].

The blast *R* gene, *Pi54*, cloned from indica rice Tetep, is of particular interest as it is one of the smallest reported blast *R* genes. *Pi54* confers broad spectrum, durable resistance against *M. oryzae* infection [95,165]. The functional role of *Pi54* in blast resistance was validated using transgenic and RNAi-mediated approach [165,166,167,168,169]. Subsequently, orthologs of *Pi54* gene; *Pi54rh* and *Pi54of,* also show broad spectrum and overlapping patterns of resistance against multiple strains of *M. oryzae* [89,170,171]. We also cloned and characterized *Avr-Pi54*; this effector interacts directly with Pi54 protein through LRR and non-LRR domain [39]. The *in silico* analysis predicted that variations in LRR domain of Pi54 and its orthologs alter their interaction with the counterpart Avr-Pi54 [89,172]. The major domain of interaction of Avr-Pi54 with Pi54 and its orthologs is the non-LRR domain. A schematic diagram depicting the molecular mechanism of *Pi54* locus-mediated resistance is given in Figure 2. The predicted model for the mechanism of action of *Pi54* consists of the inactive OsRac1 protein bound to Sti1. The OsRac1 protein is activated by GEF molecule by interacting with the RhoGEF domain. The *M. oryzae*-derived chitin, a major PAMP, induces the activation of GEF, which further triggers OsRac1. Further, the PAMP receptor, OsCERK1, transfers the signal to downstream targets through the Hop/Sti1a and Hsp90 complex, which interact with OsCERK1 through its transmembrane domain [173]. Once activated, OsRac1 recruits RACK1A, which further interacts with N-terminus of RAR1 and SGT1 proteins and regulates the ROS production by interacting directly with the N-terminus of membrane bound Rboh, an NADPH oxidase [174]. Besides, the activation of rice MAPK6-mediated signaling cascade requires OsRac1, which forms a complex with MAPK6 in rice cell extracts [175]. In the process of *Pi54*-mediated resistance, it induces the expression of various defense response genes such as callose, laccase, peroxidase, and PAL, and genes related to TFs such as Dof zinc finger, MAD box, NAC6, bZIP, and WRKY. Our further analysis of transgenic rice with *Pi54* and its wild type control plant revealed that miR815c, which targets *DR* gene *OsWAK129b,* was downregulated in transgenic plants. The upregulated miRNAs include miR164c, miR164e, miR1849, miR1854-3p, miR2925, miR396c-5p, miR396c-3p, and miR812f. We concluded that the *Pi54* gene-mediated resistance response against *M. oryzae* infection is also regulated by a set of microRNAs through both PTI and ETI pathways [176].

#### 3.2.3. Chemical Modulators

**Figure 3:**
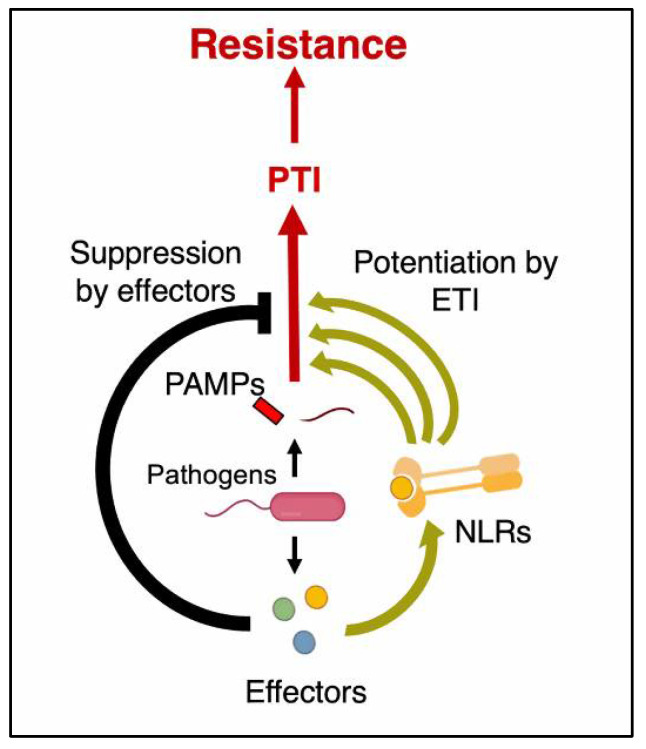
Schematic representation of the ‘Circular Model’ of plant–pathogen interaction. (This figure was originally drawn by Prof. Jonathan Jones, Sainsbury Lab, Norwich, UK, and it is being produced here with his permission).

The tug-of-war between the blast fungus *M. oryzae* and the host plant rice is an ongoing process involving various chemical molecules. In a broader sense, the general resistance response mediated by PAMP-triggered immunity and the more specific effector-triggered ETI comprise of various signaling pathways [177]. However, recently scientists stated that this model has failed to capture the stochastic process linking the initial detection of the pathogen and action of pathogen effectors, PAMPs, and damage-associated signals. The rationality of a conceptual division between PTI and ETI at a molecular or cellular level in plants has also been questioned [178]. In the view of the above observations, very recently, the famous Zig-Zag model was extended and an improved model was reported by Ngou et al. [179]. Subsequently, the new ‘Circular Model’ of plant–pathogen interaction was proposed by Yuan et al., [180], and it is also schematically represented in Figure 3. The pathways underlying the resistance response largely have a common set of overlapping defense-response mechanisms mediated by various chemical molecules. The very process of onset of resistance response, which is initiated immediately after the penetration of pathogen into rice cells, leads to atypical burst in the ROS, which is a hallmark of PTI. The ROS, which were once considered as harmful molecules for cells, are the essential components of the signaling process, leading to cell death and resistance response. Besides, ROS is also involved in the reinforcement of the cell wall during blast pathogen infection [181,182]. Other pathogen-inhibitory metabolites that have been shown to inhibit *M. oryzae* include, cyanides, Bayogenin 3-O-cellobioside, and phytoalexins. Cyanide is reported to contribute to restricting blast fungal growth [183], whereas saponin bayogenin 3-*O*-cellobioside confers cultivar-independent resistance against the pathogen [184]. Phytoalexins such as momilactone, oryzalexin, sakuranetin, and phytocassanes are reported to be induced in rice plants in response to fungal infection and have a greater activity against *M. oryzae* [185,186]. Diterpenoid gene cluster (DGC7) coding for diterpenoids, a major group of phytoalexins in rice, was characterized for its role in disease resistance [187]. The rice *DR* gene ethylene insensitive 2 (*OsEIN2*) possibly activates phytoalexin production after infection with *M. oryzae* to promote resistance [188].

Among the chemical modulators, phytohormones have their own significance when it comes to disease resistance in plants. These hormones, mainly salicylic acid (SA), jasmonic acid (JA), and ethylene (ET) have a clear dichotomy, while regulating the defense response. Against biotrophic pathogens, the regulation of immunity is mediated by SA, whereas JA and ET are the key regulators of immune response against necrotrophs and insect pests [189]. Intriguingly, rice plants challenged with *M. oryzae* and leaf blight pathogen *Xanthomonas oryzae* pv *oryzae* show no elevation in SA levels, however, the external application of SA does induce immune response [189]. Besides, at least a couple of studies indicate that *M. oryzae* manipulates the rice JA-signaling pathway and interfere with SAR [190,191]. In one study, *M. oryzae* was found to suppress transcription factor gene *OsTCP21*, a positive regulator of blast resistance, through targeted induction of miR319 [190]. Hence, *M. oryzae* could reduce the biosynthesis of JA in rice via inducing rice miR319. Secondly, *M. oryzae* modifies the rice active JA molecule to an inactive 12-OH JA, thereby denting the host-resistance response [192]. In rice, infection with *M. oryzae* represses the auxin signaling pathway in the distal healthy leaves by repressing the auxin-responsive genes such as *ARF1* and *IAA9* and thereby inducing systemic acquired resistance (SAR) against this pathogen [193]. On the contrary, the accumulation of auxins leads to rice susceptibility to *M. oryzae* [194]. Similar to auxins, cytokinins (CKs) were also found to be induced upon *M. oryzae* infection. Defense response genes *OsPR1b* and *PBZ* were induced by the CKs accumulated post-*M. oryzae* infection, and this resistance response is in synergy with the SA signaling pathway [195].

**Figure 4:**
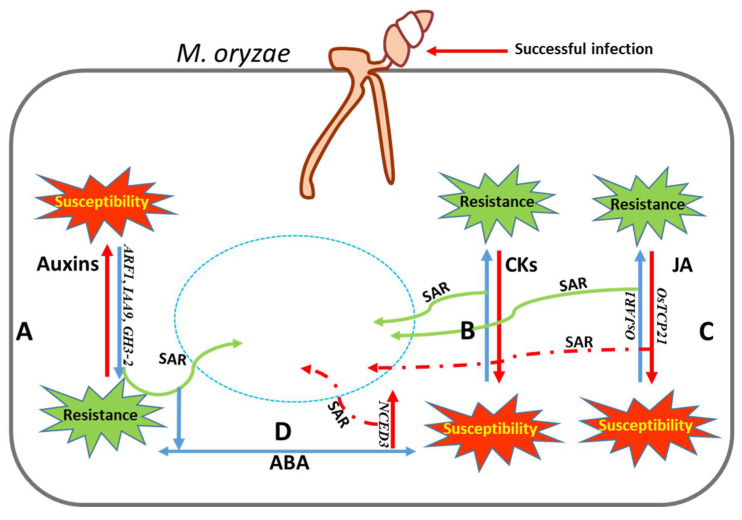
Representation of role of phytohormones in rice during *M. oryzae* infection. (**A**) Auxin: Upon infection with *M. oryzae*, rice reduces the production of auxin and thereby induces SAR. However, the pathogen secretes auxins to counteract this host-induced SAR. (**B**) Cytokinin: The host CKs at higher concentration induce SA-mediated SAR. (**C**) Jasmonic acid: JA through induction of *OsJAR1* induces resistance, whereas *M. oryzae* counteracts this through activation of *miR319,* which suppresses the expression of rice *OsTCP21* and blocks the SAR-induced resistance. (**D**) Abscisic acid: ABA generally has antagonistic effects on blast disease resistance. *M. oryzae* induces the expression of rice *NCED3* gene and thereby ABA biosynthesis and increased susceptibility to pathogens by inhibiting SAR.

Unlike auxins and CKs, ABA has an antagonistic effect on disease resistance through the suppression of SAR mediated by SA, JA, and ET signaling pathways [196]. It was observed that a reduction or disruption of ABA signaling enhanced blast resistance, whereas exogenous ABA application enhanced the susceptibility towards *M. oryzae* in rice [193,197]. A schematic diagram depicting the role of reported phytohormones in rice blast disease response is presented in Figure 4.

#### 3.2.4. Modulation of Coding RNA (mRNA) of Rice upon *M. oryzae* Infection

The *R* gene-mediated resistance in rice blast is well established. So far, eight *R* genes (*Pi9, Pi54, Pigm, Pi50, pi21, Pi7, Pi57,* and *Ptr*) have shown to confer broad spectrum blast resistance. Defense response (*DR*) genes also generally mediate broad-spectrum resistance to pathogens. Till now, seven *DR* genes or factors have been identified that positively and negatively regulate blast resistance. These *DR* genes mainly belong to TFs and kinases. *R* and *DR* genes work in concert to induce resistance against *M. oryzae* [91]. Several other co-operations may exist among *R* and *DR* genes but are still to be explored. The information on prevalent race flora of *M. oryzae* is required to stack the correct *R* genes in rice lines. In the absence of this information, stacking *R* genes together with *DR* genes may prove more useful for better disease management [91]. Transcriptome of rice upon *Magnaporthe* infection at different time points has been studied extensively in different rice genotypes [198]. These studies mainly reveal the expression levels of different coding transcripts and their probable functions in different temporal and spatial conditions. The compatible and incompatible interactions of *M. oryzae* with different rice lines (resistant or susceptible) lead to the expression of different sets of coding transcripts.

The majority of studies on rice transcriptomes upon *M. oryzae* infection provide a comparative picture of changes in expression level of *DR* genes in compatible and incompatible interactions. Transcriptome studies of rice NILs differing in a single-blast *R* gene give a clear picture to understand the mechanism of *R–Avr* and *DR* gene-mediated resistance. Comparative studies of NILs carrying different blast resistance genes (*Pi9*, *Pi54* and *Pi1*) compared to susceptible control have shown that the transcripts related to cell wall structure (suberin biosynthesis) and secondary metabolite (JA biosynthesis, salicylate biosynthesis, 13-lox and 13-hpl, divinyl ether biosynthesis, and phenylpropanoid biosynthesis) were differentially expressed in all three NILs upon *M. oryzae* infection [199]. The study revealed that the NILs containing different blast resistance genes in a common background have some common set of functionally important transcript whose expression changes upon *M. oryzae* infection. Similarly, each NIL had a unique set of transcripts that change their expression upon blast pathogen infection. Further, at 24 hpi with *M. oryzae,* a significant change was recorded in the expression of genes related to biosynthesis of trehalose, flavonoids, aminopropanol, cellulose, UDP-D glucarate, xylose, serine and choline, cyclopropane FA and Cyclopropene FA, phaseic acid, and phytocassane. Whereas at 72 hpi, a significant change in the expression levels of genes involved in triacylglycerol biosynthesis, flavonoids biosynthesis, GDP-mannose metabolism, trehalose biosynthesis-I, stachyose biosynthesis, mannose degradation, and nitrate reduction was recorded [199,200].

Transgenic lines help in understanding the basic mechanism of disease resistance by over expressing the genes responsible for disease resistance. During early stages of infection, rice cell wall offers the first level of a physical barrier to *M. oryzae* appressorium. So, genes related to cell wall modification show significant change in their expression upon pathogen infection. Callose (1, 3-β-glucan) and lignin are important components of the plant defense response and are involved in the blockage of plasmodesmata, thus posing a physical barrier for the penetration of fungal mycelia into nearby plant cells [198]. Two genes coding for callose biosynthesis were very highly upregulated in transgenic rice line TP-Pi54 in comparison to non-transgenic control lines upon challenge with *M. oryzae* [182]. In the case of *Pi54*, the role of callose in rice blast resistance has already been reported [165].

During the early stages of infection, enzymes such as beta-glucanase and chitinase enhance the resistance against fungus by catalyzing the hydrolytic cleavage of glycosidic linkages in β-glucans present in the fungal cell wall [201]. In rice, it was reported that beta-glucanase is highly induced in response to *M. oryzae* infection [202,203]. In the incompatible interactions [174,182], class III peroxidase gene was found to be highly up-regulated after blast pathogen infection [182,200]. Peroxidases are found to be involved in lignin biosynthesis [204] and cross-linking of cell-wall components, thus fortifying the cell wall against pathogen infection. Redoxin, glutathioredoxin, peroxidase, and catalase are major ROS-scavenging enzymes that fine tune ROS signaling and restrict ROS-dependent damage [205]. In both compatible and incompatible interactions, changes in the expression level of genes involved in cell wall biosynthesis, cell wall modulation, and cell wall degradation have been observed with a number of upregulated genes being higher in incompatible interaction compared to compatible interaction [198]. The studies suggest that during the initial phase of interaction with *M. oryzae*, rice plants enforce the defense mechanism by cell wall fortification in both resistant and susceptible rice lines [198]. Once the primary defense mediated by cell wall reinforcement is breached, the next level of defense response is induced. Transgenic rice line *Pi21*-RNAi showed changes in the expression levels of 43 receptor kinases (belonging to WAK, L-LEC, LRR, CAMK, MEKK, LRK10L-2, SD, DUF26, RLCK, and CR4L subfamilies) associated with PAMP recognition and calcium ion influx [206]. These kinases are involved in perceiving internal and external signals. As a result, a more robust PTI was observed in the *Pi21*-RNAi line compared to susceptible control, and 53 TF genes (WRKY, NAC, DOF, and ERF families) were observed to be differentially expressed in the *Pi21*-RNAi line. Cell wall-associated kinases participate both positively and negatively in basal defense against rice blast fungus [207]. The BAK1 is a coreceptor of receptor-like kinase RLKs [208] and acts as a central regulator in PTI [209]. During the later stages of fungal infection, MAPK (mitogen-activated protein kinase) cascades play an important role in downstream signaling processes leading to ETI. Receptor kinase and MAPK act as signaling molecules in both PTI and ETI [198,199,200]. As a part of ETI, several TF genes are induced. *WRKY* genes are one of the major families among them, and many of them such as *WRKY76*, *WRKY47*, *WRKY45*, *WRKY55*, *WRKY53*, *WRKY62*, and *WRKY71* are induced in rice upon *M. oryzae* infection [201,210,211]. Similarly, differential expression of 53 TFs such as *WRKY*, *NAC*, *DOF*, and *ERF* families, and 62 *PR* genes was recorded between *Pi21*-RNAi and Nipponbare [51]. The role of WRKY genes in the activation of several PR genes was also reported [210,212]. A higher number of WRKY genes is reported to be upregulated in blast-resistant genotype GV (WRKY 45, WRKY79) and near-isogenic line-carrying blast-resistance gene *Pi9* compared to susceptible control [213,214].

Phytohormones play a significant role during compatible and incompatible rice–*M. oryzae* interactions. JA–ET and SA pathways act synergistically in pathogen-triggered immunity (PTI) response, while ETI uses the JA-ET pathway when SA signaling is not present in the plant [215]. Several reports confirm that changes in JA expression act as a powerful mediator of resistance against *M. oryzae* [216,217]. A high upregulation of genes involved in ET and JA metabolism has been reported in incompatible rice–*M. oryzae* interactions [6,199]. A common upregulation of transcripts involved in JA biosynthesis was observed in NILs carrying blast-resistance gene *Pi9*, *Pi54,* and *Pi1* following *M. oryzae* inoculation [199]. A higher level of upregulation of lipoxygenase (Lox), a gene involved in JA biosynthesis, was reported in resistant lines compared to susceptible control [51,199,200]. Similarly, an upregulation of genes related with ethylene, salicylic acid (SA), and jasmonic acid (JA) signaling was reported in incompatible interactions involving *M. oryzae* [203].

Genes governing secondary metabolism are another set of genes that are upregulated in resistant and susceptible rice lines after *M. oryzae* infection. A large number of enzymes that are part of phenylalanine and shikimate biosynthesis as well as downstream phenylpropanoid biosynthesis show upregulation in resistant *Pi9* NILs [199,214] and transgenic lines carrying the *Pi54* gene [182]. The phenylpropanoid pathway plays an important role in the rice–*M. oryzae* interaction, because phenylpropanoids are important antimicrobial compounds. This pathway is involved in synthesizing lignin and phytoalexins that prevent pathogen invasion of the host cells. Rice plants accumulate diterpene, phytoalexins, phytocassanes, oryzalexins, and momilactones to counteract *M. oryzae* invasion [218,219]. The important genes involved in the biosynthesis of diterpene phytoalexin, momilactone, and phytocassanases are reported to be upregulated in the blast-resistant genotype after *M. oryzae* infection [213]. The diterpene phytoalexin biosynthetic (DPB) gene *OsKSL8* (LOC_Os11g28530; oryzalexin S synthesis) shows higher expression in the resistant than in the susceptible genotype after *M. oryzae* infection [213]. A higher accumulation of phytoalexins Momilactone A and Sakuranetin has been observed in incompatible compared to compatible interactions [213]. These phytoalexins inhibit germination of *M. oryzae* spores in infected leaves [220,221,222], thus suggesting the importance of genes of diterpene phytoalexin biosynthesis in resistance to *M. oryzae.*

A comparison of the rice–*M. oryzae* interaction transcriptome from our laboratory with previous six studies indicated that the cytochrome *P450* gene was commonly upregulated in all these studies. We also observed that the transcriptome of rice varies quantitatively rather than qualitatively during incompatible and compatible interactions with functional categories of differentially expressed genes remaining similar in both the cases [198].

#### 3.2.5. Small and Long Non-Coding RNA Play a Regulatory Role in Rice upon *M. oryzae* Infection

Small non-coding RNA include microRNA (miRNA), small interfering RNA, piwi-interacting RNAs, trans-acting siRNAs, and natural antisense transcript siRNAs. In rice, miRNAs are well known to control many important agronomic traits such as tiller development, flowering time, panicle establishment, grain formation, and yield production, and also regulate a plant’s response to both abiotic and biotic stresses [223,224]. Few conserved novel miRNAs and miRNAs families have been found to be responsive to blast fungal elicitors, which suggests their possible involvement in rice–*M. oryzae* interaction [225]. Among eight rice *DCL* genes, till now only *OsDCL1a* has been studied and was found to negatively regulate PTI against *M. oryzae,* while the knock-down of *OsDCL1a* enhanced resistance to the blast disease [226]. Four miRNA families, namely miR160, miR166, miR398, and miR7695 have been reported to act as positive regulators of rice immunity against *M. oryzae* [227]. *OsDCL1s* is known to act as a key molecule in the miRNA signaling pathway that mediates cross talk between rice-*M. oryzae* interaction and miRNA network. Our group recently deciphered the role of miRNAs in *Pi54* mediated incompatible interaction with blast pathogen. Upregulated expression levels of miR164c, miR164e, miR1849, miR1854-3p, miR2925, miR396c-5p, miR396c-3p, and miR812f have been found in the resistant line in comparison to susceptible control [182].

### 3.3. Interplay between Rice-M. oryzae: A Classical Example for Plant-Pathogen Interactions

**Figure 5:**
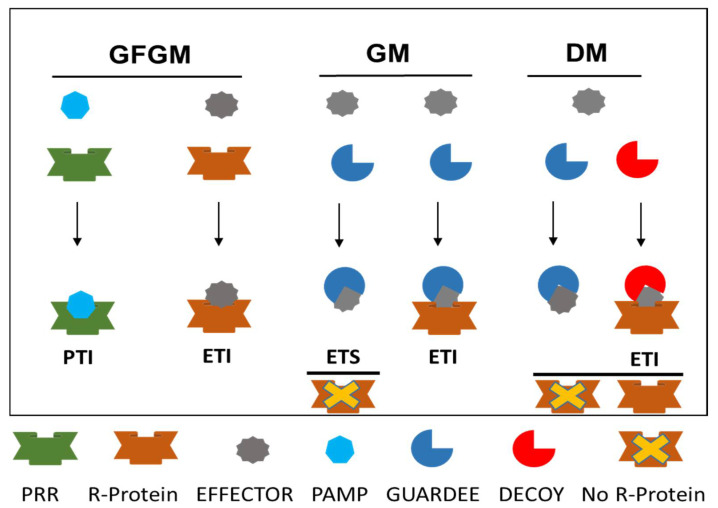
Simplified diagrammatic representation of models on host disease-resistance mechanism. Gene for gene model (GFGM): Physical interaction of pathogen-derived PAMP (pathogen associated molecular pattern) with DR protein (pattern recognition receptor, PRR), resulting in PAMP-triggered immunity (PTI); physical interaction of Avr (effector) with R protein (NBS-LRR), resulting in effector-triggered immunity (ETI); Guard Model (GM): Interaction of effector with guardee triggers effector-triggered immunity (ETI) by their interaction with R protein (NBS-LRR). However, in the absence of R protein, binding of guardee with effector enhances the susceptibility/fitness of the pathogen (ETS); Decoy Model (DM): Interaction of effector with decoy triggers effector-triggered immunity (ETI) with the interaction of R protein (NBS-LRR), however, in the absence of R protein, there is no increase in the virulence/fitness of the pathogen.

Antagonistic interactions between rice plants and the fungus, *M. oryzae*, result in ‘arms races’. While the plant attempts to recognize the pathogen and subvert its growth and spread, the pathogen tries to subvert recognition and suppress the host responses [228]. Several hypothesis and models were proposed to explain the race that underpins disease resistance. We discuss here three models: Gene-for-gene (GFGM) model, Guard model (GM), and Decoy model (DM), with diagrammatic representation (Figure 5).

#### 3.3.1. Gene-for-Gene Model

The gene-for-gene concept, initially discovered in the flax-rust pathosystem by Harold Henry Flor [229], essentially describes an interaction between a matching gene in a host (called resistance *R* gene) and pathogen (called avirulence or *Avr* gene) that interact physically in a receptor–ligand mode to initiate the defense response. *R*-genes and their structure were described earlier [230,231]. The majority of the *R*-genes harbor signature nucleotide binding site—leucine rich repeat (NBS-LRR) or pattern recognition receptors (PRR). *Avr,* on the other hand, can be *effectors*, the pathogen-secreted proteins that manipulate host cell functions, or molecular motifs conserved across many pathogen species known as pathogen-associated molecular patterns (PAMPs). LRRs (approximately 24 amino acid motifs) are principally involved in protein–protein (R–Avr) interaction, and the greatest variation in this class emanates from the LRR domain. The classic examples of R–Avr interaction include pairs of R–Avr proteins such as Pi54 and AvrPi54 [39], and Pi-ta and Avr-Pita [184]. PRR, on the other hand, recognize generic motifs of the pathogen and initiate reaction, e.g., rice *Xa21*-resistance gene recognizes the tyrosine-sulphated protein, RaxX [232]. Currently, more than a dozen examples of direct physical interactions are available, and interestingly, other domains are reported to interact physically to initiate defense response [89].

#### 3.3.2. Guard Model

The Guard Model envisages an indirect physical interaction between R and effector proteins. The model predicts that R proteins act by monitoring/guarding the indispensable effector target (called *guardee*) and any modification of this target by the effector results in the activation of the R protein [233]. This indirect perception could explain how multiple effectors could be perceived by a single R protein, thus enabling a handful of *R* genes to provide protection against a great variety of pathogens. Classical examples of the hypothesis are tomato RCR3 and Pto and *Arabidopsis* RIN4 and PBS1 [133,234]. In rice, three gene pairs coding for CC-NLRs are understood to act in a Guard Model. These genes include *RGA4/RGA5*, *Pik-1/Pik-2*, and *Pi5-1/Pi5-2* [44,235,236,237,238]. The proteins RGA4/RGA5 is necessary for AVR-Pia- and AVR1-CO39-induced and Pia- and Pi-CO39-mediated blast resistance in rice. Similarly, Pikh-1 proteins act as guardees and perceive AvrPik-h protein, which further induces Pikh-2-mediated resistance response.

#### 3.3.3. Decoy Model

The Decoy Model envisages that the arms race of host–pathogen interaction leads to the evolution of ‘decoys’, which mimics effector targets to trap the pathogen into a recognition event, but itself has no function either in the development of disease or resistance. Decoys might evolve from effector targets by two ways: (a) gene duplication and evolution and (b) mimicking effector targets (target mimicry) [177,234]. The current understanding suggests that these interactions drive the evolution of ‘decoys’ in pathogens as well. Interestingly, decoys undergo a similar manipulation as the component they mimic, but play an opposite role, either by preventing manipulation of the component they mimic or by triggering a molecular recognition event. Three different types of decoy are reported: (a) Receptor decoys: mimics to absorb ligands, (b) bodyguard decoys: protecting secreted virulence factors, and (c) sensing decoys: mimics effector targets acting as coreceptors with two different modes: sponge and bait [239]. Currently, there are few cases illustrating decoy mechanisms, however, much remains to be discovered. The discovery of more decoys shall enhance our understanding of disease resistance and provide a means to improve host immunity e.g., decoy engineering of PBS1 in *Arabidopsis* plants confers a broader resistance spectrum [240]. Interestingly, a putative decoy protein, zinc finger–BED type (ZBED), was investigated for its role in rice resistance and susceptibility to the blast fungus [241]. Similarly, the RATX1 domain of RGA5 acts as a decoy domain for recognizing the *M. oryzae* effector AVR-Pia [242].

## 4. Resistance Response of Rice to Blast Disease

### 4.1. Resistance Response Based on Quantitative Trait Loci (QTL)

Resistance to rice blast in some cases is known to be inherited as quantitative trait regulated by many genes. Contemporary approaches that are employed for other polygenetic traits are also applicable for studying the quantitatively inherited blast resistance. The basic QTL mapping approaches such as single marker analysis (SMA), simple interval mapping (SIM), and interval mapping approaches (IM) have been extensively used for detecting major and minor QTLs linked with complete/partial resistance to rice blast [108,243]. Mapping genomic regions for quantitative blast resistance has gained importance due to race non-specificity of the resistance, which provides stable protection against the pathogen [244]. For the first time, blast-resistance QTL was identified in a widely grown African variety, Moroberekan [143]. There are also instances in which the blast resistance has been shown to be regulated by race-specific resistance genes such as *Pi34*, *Pif*, *Pi21*, and *Pb1*. [146,245,246,247,248]. To date, more than 500 QTLs controlling resistance to blast disease in rice have been identified and mapped on different rice chromosomes [91]. Most of these QTL were identified from bi-parental populations derived from *japonica* and *indica* subspecies, with the assistance of various marker systems such as RFLP, SSR, and SNP [249]. The QTLs exhibiting modest individual effect with race non-specific or broad-spectrum resistance provide durable resistance [250]. Thus, it is important to identify and use novel QTLs with broad spectrum resistance for the development of resistant cultivars.

### 4.2. Resistance Gene Mediated Resistance

#### 4.2.1. The Blast Resistance Genes Identified, Mapped, and Cloned in Rice

**Figure 6:**
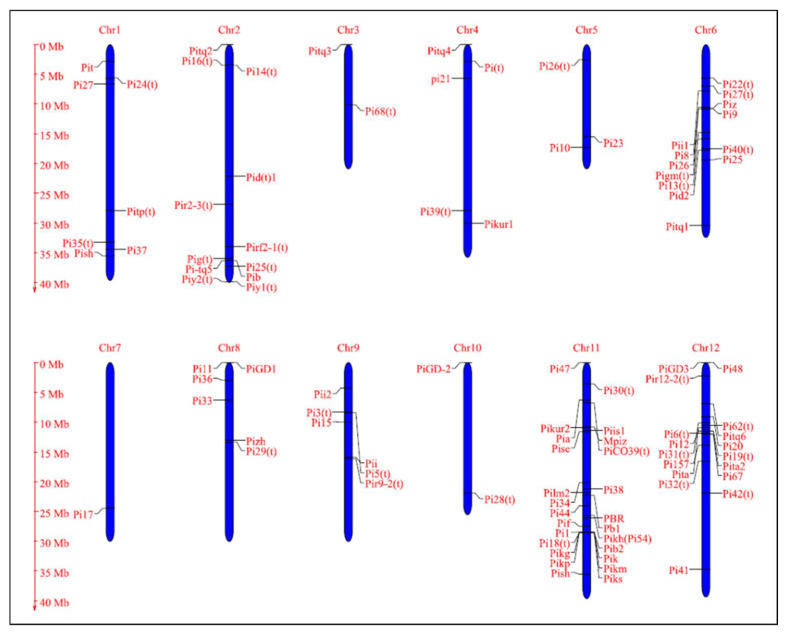
Rice blast resistance genes identified and mapped on to different rice chromosomes.

**Figure 7:**
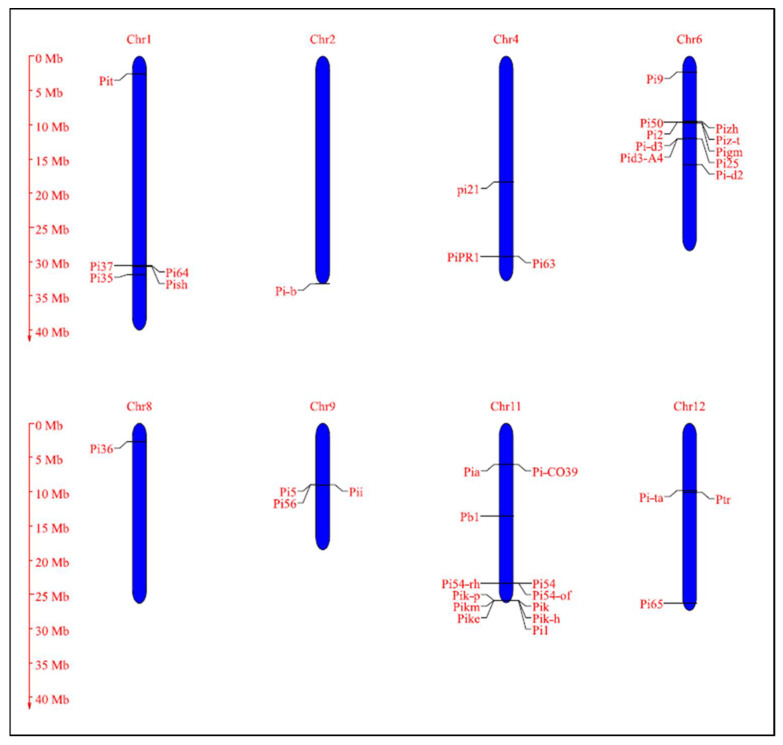
Cloned and characterized blast resistance genes on different chromosomes.

Deploying host plant resistance for disease management is an eco-friendly and most viable approach to manage the disease as a wide range of resistance alleles are available in diverse rice germplasms [123]. In rice, nearly 100 rice blast resistance genes have been identified and mapped in rice till date (Table 3, Figure 6) [3]. Among the identified resistance genes, 38 have been cloned and are known to encode proteins with nucleotide-binding sites (NBS) and leucine-rich repeat (LRR) domains (Table 4) [101,251]. Exceptions include *Pi-d2*, which encodes a B-lectin kinase domain protein, *pi21* that encodes a proline-rich protein with a heavy metal domain, and *Ptr* that encodes an atypical protein with an armadillo repeat [4,101]. *Pik*, *Pikm*, *Pik-p*, *Pi1*, *Pike*, *Pi5*, *Pia,* and *Pi-CO39* contain two NBS-LRR protein structural genes for blast resistance. *Pi5-1*, *Pb1*, *pi21,* and *Pi63* genes are induced by pathogen infection, while the remaining genes express constitutively. The majority of the cloned *R* genes induce resistance against leaf blast at the seedling stage, while only a few *R* genes, such as *Pb1*, *Pi25*, *Pi64,* and *Pi68* confer resistance to panicle blast [252,253,254,255]. Most of the identified blast resistances are clustered on chromosomes 6, 11, and 12 (Figure 6), while cloned blast resistance genes are clustered on chromosome 6 and 11 (Figure 7). Notably, a large number of *R* genes (28 *R* genes) are mapped on to chromosome 11, and chromosomes 3, 7, and 10 carry solitary *R* genes, respectively. The genes *Pi2, Pi54, Pi9, Pigm,* and *Pizt* mapped on to chromosome 6 are known to provide broad spectrum resistance, and so is *Pi54,* which is mapped on to chromosome 11. A great majority of blast resistance genes are distributed into clusters of tightly linked genes. At least three major clusters of blast resistance genes have been detected in rice on chromosomes 6, 11, and 12 (Figure 6). Several studies have demonstrated that the genetic control of blast resistance is complex and involves both major and minor genes with additive/complementary interactions [256]. To be ahead in the evolutionary race between pathogen and host, there is a need for continuous identification of new *R* genes from the previously untapped genetic resources [177].

**Table 4:** The cloned and functionally characterized rice blast resistance genes.

Genes & Alleles	Encoded Protein	Chr. No	Cognate *AVR* Gene	Chromosomal Location	Donor	Reference
*Pish*	NLR	1	-	33,136,846–33,145,541	Nipponbare	[257]
*Pi35*	NLR	1	-	33,838,140–35,206,760	Hokkai 188	[111]
*Pi37*	NLR	1	-	33,116,117–33,124,371	St. No. 1	[112]
*Pi64*	NLR	1	-	33,098,072–33,104,550	Yangmaogu	[254]
*Pit*	NLR	1	-	2,686,729–2,687,700	K59	[258,259]
*Pi-b*	NLR	2	*AVR-Pib*	35,979,234	Tohoku IL9	[38,118]
*pi21*	Proline-rich metal binding protein	4	-	19,836,301–19,835,131	Owarihatamochi	[96]
*Pi63*	NLR	4	-	31,553,065–31,558,406	Kahei	[260]
*PiPR1*	NLR	4	-	316,00,121–31,604,201		[261]
*Pi9*	NLR	6	*AVR-Pi9*	2,410,176–2,418,568	75-1-127	[37,133]
*Pi2*	NLR	6		1,043,5816–10,441,907	Jefferson	[262]
*Piz-t*	NLR	6	*Avr-Pizt*	10,387,509–10,390,465	Zenith	[36,262]
*Pi50*	NLR	6	-	10,375,846–10,380,263	Er-Ba-zhan (EBZ)	[97]
*Pizh*	NLR	6	-	10,087,244–10,478,622		[263]
*Pigm*	NLR	6	-	Near to 10,435,816–10,441,907	Gumei4	[100,264]
*Pi-d2*	B-lectin receptor kinase	6	-	17,164,851–17,160,330	Digu	[137]
*Pi-d3*	NLR	6	-	13,058,027–13,055,162	Digu	[265,266]
*Pi25*	NLR	6	-	13,058,027–13,055,162 (*Pid3* allele)	Gumei2	[253]
*Pid3-A4*	NLR	6	-	13,058,027–13,055,162 (*Pid3* allele)	A4 (*Oryza rufipogon*)	[267]
*Pi36*	NLR	8	-	2,878,953–2,890,634	Kasalath	[268]
*Pi5*	NLR	9	-	9,674,695–9,674,000	RIL260	[236]
*Pii*	NLR	9	*AVR-Pii*	9,674,695–9,674,000	Hitomebore	[16,269]
*Pi56*	NLR	9	-	9,777,527–9,780,698	Sanhuangzhan No. 2	[270]
*Pb1*	NLR	11	-	14,705,215–14,714,572	Modan	[252,271]
*Pik*	NLR	11	*AVR-Pik*	27,984,697–27,989,134	Kusabue	[16,272]
*Pik-p*	NLR	11	*AVR-Pikp*	27,978,568–27,980,621	K60	[237]
*Pikm*	NLR	11	*AVR-Pikm*	27,984,697–27,989,134	Tsuyuake	[235]
*Pike*	NLR	11	-	27,984,697–27,989,134 (*Pik* allele)	Xiangzao143	[273]
*Pik-h*	NLR	11	-	27,984,697–27,989,134 (*Pik* allele)	K3	[238]
*Pi1*	NLR	11	-	27,984,697–27,989,134 (*Pik* allele)	C101LAC	[274]
*Pi54*	NLR	11	*AVR-Pi54*	25,262,834–25,264,520	Tetep	[39,275]
*Pi54rh*	NLR	11	*Avr-Pi54*	25,262,834–25,264,520 (*Pi54* allele)	*Oryza rhizomatis* (nrcpb 002)	[171]
*Pi54of*	NLR	11	*AVR-Pi54*	25,262,834–25,264,520 (*Pi54* allele)	*Oryza officinalis* (nrcpb004)	[89]
*Pia*	NLR	11	*AVR-Pia*	6,546,026–6,541,924	Sasanishiki	[16,44,276]
*Pi-CO39*	NLR	11	*AVR-CO39*	6,888,057-6,291,466	CO39	[142,277]
*Pi-ta*	NLR	12	*AVR-Pita*	10,612,068–10,606,359	Yashiro-mochi	[32,162]
*Pi65*	LRR-RLK	12		28,376,327–28,379,731	GangYu129	[278]
*Ptr*	ARM repeat domain protein	12	-	10822534–10833768	M2354	[101]

NLR, nucleotide-binding leucine-rich repeat; AVR, avirulence; Chr. No, Chromosome number.

#### 4.2.2. Resistance Response Mediated by Alleles of Known R Genes

The deployment of cultivars introgressed with resistance (*R)* genes is most effective and eco-friendly approach to control blast disease [4]. Among the 38 cloned and characterized blast *R* genes (Table 4), except for *pi21*, which is a recessive *R* gene, the remaining 37 are dominant genes. Most of the cloned blast resistance genes are alleles of the previously cloned rice blast *R* genes and few of them represent a new rice blast *R* locus [260,279]. Considering that more than 400 *NBS–LRR* gene sequences are identified in a rice genome and alleles of rice blast *R* genes may confer distinct resistance spectra to *M*. *oryzae* isolates, allele mining of cloned rice blast *R* genes in rice germplasms would reveal more favorable *R* alleles for rice blast resistance breeding [89,280]. TILLING (Targeting Induced Local Lesions in Genomes) and PCR based mining are the two main approaches for the identification of better, new and superior alleles of disease resistance genes [281,282]. Natural mutations such as transition, transversion, point mutation, and insertion, and deletions (InDels) are the main driving forces for the generation and evolution of new alleles. With the availability of enormous database information, desired and superior alleles can be easily identified and retrieved [282]. The allele mining approach identifies new haplotypes and evolution patterns of *R* genes [282]. The superior allelic variants with novel resistance specificities can be deployed in breeding programs to achieve broad spectrum resistance to blast.

Many studies have reported allele mining of blast resistance genes from wild and cultivated species of rice [170,283,284,285,286]. Studies of *Pi-ta* gene in wild (AA and CC genome) and cultivated species of rice indicated a consensus conserved sequence before divergence [287]. In another study, *Pi-ta* orthologs identified in 26 rice accessions from 10 different countries were shown to display a dimorphic pattern of nucleotide polymorphism and low nucleotide diversity at the LRD region of the orthologs [16]. To study allelic variants and flanking sequence of *Pi-ta,* 159 geographically diverse accessions of *Oryza* species (AA genome) have been used [288]. The *Pi-ta* and *Pi9* alleles have been studied extensively in Indian landraces [289,290]. Five unique and novel *Pi-ta* variants were identified from local landraces of rice. Notably, strong selective sweeps as indicated by the high value of Pi (non/syn) on the LRD were inferred to shape the evolution of the new alleles at *Pita* locus [290]. Other blast resistance loci such as *Pid3 and Pi9* have been explored to study the nucleotide polymorphism and evolutionary pressure [265,291]. A nucleotide polymorphism study of the *Piz-t* locus of Indian landraces indicated positive selection pressure on the locus and the role of diversification of the LRR domain in the evolution of a gene [292]. PCR-based allele mining for blast resistance gene *Pi54* from six cultivated rice lines and eight wild rice species was undertaken to understand its structural variation and its impact on the phenotypes. A high nucleotide variation was recorded between cultivated and wild species (35–90%) compared to variation in cultivated species (1–20%) [177]. The *Pi54* allele mining in 92 rice lines indicated an extensive variability in the allelic sequences and unique haplotypes linked to resistance alleles. InDel polymorphisms in the allelic variants have been targeted for developing markers for the identification of better allele(s) and their introgression in commercial rice cultivars, employing marker-assisted selection [293]. Similarly, the allelic diversity of *Pi54* gene has also been studied in 885 Indian rice laces that have shown resistance to naturally existing pathogens as well as against 5 unique strains of the blast pathogen. Nine new alleles of *Pi54* were identified based on the sequence comparison to the *Pi54* reference sequence as well as to already known *Pi54* alleles [279]. Allelic mining of the blast resistance *Pid3* locus in 3000 rice genome project (3 K RGP) genomes revealed that most *japonica* rice accessions harbored pseudogenes due to premature stop mutations, while *Pd3* alleles in most of the *indica* rice accessions were identical to the functional haplotype, which had a similar resistance spectrum as the previously reported *Pid3* gene [294]. In another study, 13 novel alleles of *Pi9* were identified based on tandem-repeat regions from 361 resistant rice varieties [295].

## 5. Molecular Mechanisms of Leaf and Panicle Blast

Blast infects the rice crop at all stages of its growth, starting from the nursery to the grain filling stage, under favorable environmental conditions. Blast pathogen likes leaf wetness, high humidity, longer free moisture periods, a night temperature between 18–24 °C, and an absence of wind at night time. Fungal spores are produced and spread under high relative humidity conditions, however, no spore production is observed below 89% relative humidity. The sporulation process increases with relative humidity above 93%. The most appropriate temperature for spore germination, lesion formation, and sporulation is 25–28 °C [296].

Of the two commonly recognized phases of the disease, the leaf blast occurs during the plant’s vegetative stage, while the neck blast (a near synonym of panicle blast) appears during the reproductive stage. The flow of photosynthates to growing grains is blocked at the base of the panicle during neck blast infection, resulting in chaffy grains or empty panicles. Under epidemic conditions, the damage inflicted by neck blast infection could be twice as severe as leaf blast with losses approaching up to 70% of the anticipated yield [297]. Although more than 100 *R*-genes for leaf blast resistance are known, very few genes for resistance to neck blast have been identified and located on the rice genome. The disease response of leaf and panicle to blast infection is different, and the varieties that are susceptible to leaf blast are resistant to neck blast and vice versa [297,298]. Some of the cultivars resistant at the seedling stage become susceptible to neck blast [299]. The reported susceptibility of leaf blast resistance genotypes to neck blast and vice versa has suggested that the different genes are involved in resistance to leaf and neck blast [297,300]. In other studies, the gene/QTLs for panicle blast resistance were mapped to the genomic locations harboring major leaf blast resistance genes, thereby suggesting the existence of common genes for resistance to both phases of the disease [301]. *Pb1* gene mapped on the long arm of chromosome 11 in an *indica* cultivar ‘Modan’ is the first panicle blast resistance gene to be identified from rice [147]. The gene was introgressed into several varieties in Japan and has shown durable resistance to blast for almost 30 years [252]. The gene exhibits lower expression levels at the seedling stage but its expression reaches peak during full-heading stage, thus accounting for its strong resistance to panicle blast. These findings have been taken to reflect that the temporal and spatial expression pattern of a blast resistance gene is a major factor in deciding whether the gene will offer protection to leaf or neck blast or both phases of the disease [302]. Zhuang et al. [156] identified a blast resistance gene *Pi25(t)* that provides resistance to both leaf and neck blast. Ma et al. [254] identified a resistance gene *Pi64,* which is constitutively expressed in all the tissues and provides protection against both leaf and neck blast. Noenplab et al. [301] reported co-localization of QTLs for leaf and neck blast resistance on the same genomic regions on chromosomes 1, 11, and 12.

Ishihara et al. [303] identified a major QTL, *qPbm11*, for panicle blast resistance in the genomic region on chromosome 11, from where panicle blast resistance locus, *Pb1,* has previously been identified in cultivar Modan. However, the absence of *Pb1*-encoded transcripts in the panicles of *qPbm11* genotype Miyazakimochi has suggested that the *qPbm11* is different from *Pb1.* Fang et al. [304] identified a panicle blast resistance QTL, *qPbh-11–1*, located on the long arm of chromosome 11. The gene occupies a different genomic position compared to two panicle blast resistance loci *Pb1* and *qPbm11* previously identified from the same chromosome. The gene expression studies have suggested that the genes such as *Pb1* that are expressed during the heading stage shall display neck and panicle blast resistance, while those showing constitutive expression such as *Pi64* are expected to provide protection against both the phases of the disease. These studies have provided a plausible explanation for observed inconsistencies in the reaction of leaf blast resistance varieties to neck blast and vice-versa, which were previously ascribed solely to either shifts in race composition of the pathogen or changes in environmental conditions during the crop season.

## 6. Management of Blast Disease Using Host Resistance

### 6.1. Introgression of QTLs for Blast Resistance

The introgression of *R* and QTLs genes in rice is considered to be one of the best ways to control blast disease [305]. Therefore, deploying single or multiple QTLs that impart the partial resistance and non-race specificity is a priority in rice breeding nowadays [306,307]. Though there are more than 500 reported QTLs for blast resistance, only few of them have been deployed under field conditions. There are various reports in which QTLs have successfully been transferred in rice to achieve broad spectrum resistance against rice blast disease. Pyramiding of resistance QTLs in cultivated varieties has been practiced to increase disease resistance levels, e.g., two QTLs (*qBl1* and *qBl11*) identified from the rice cultivar Jao Hom Nin (JHN) were introgressed into the Thai glutinous jasmine rice cultivar RD6 MAB [308]. Using the MAS approach, elite indica rice lines were developed by bringing together multiple QTLs from IR64 and JNJ into a single background, and resulting lines have shown broad spectrum resistance against Thai blast isolates [309]. A new glutinous rice variety was developed by pyramiding *Sub1*, *badh2*, *qBl1*, and *qBl11* loci from the rice lines IR85264 (*Sub1*), TDK303 (*badh2*), and RGD07529 (*qBl1*+*qBl11*) into a single background [310]. Further, Fukuoka et al. [311] combined the partial resistance genes and QTLs (*pi21*, *Pi34*, *qBR4-2*, and *qBR12-1*) to enhance the blast resistance in rice. Suwannual et al. [312] used four blast resistance QTLs from two rice lines for the development of pyramided broad spectrum blast resistance rice lines of a popular variety RD6. The RD6 introgression lines carrying a high number of QTLs displayed broad-spectrum resistance to prevalent blast pathogen races. Recently, the introgression of a major QTL *qBL3* for leaf and neck blast resistance into a susceptible rice variety BPT5204 resulted in progeny lines showing field resistance to leaf and neck blast [123].

### 6.2. Introgression of R-Genes for Blast Resistance

Of the various means available to curb the blast disease, breeding resistance varieties is the most suitable, ecologically safe, and cost-effective strategy. Since the resistance to blast in rice involves gene-for-gene interactions, the varieties carrying single *R* genes often succumb to disease due to the appearance of virulent races of the pathogen, which is due to the mutation of avirulence gene to evade detection by the corresponding host *R*-gene [313]. Pyramiding of multiple *R*-genes has been advocated to foster enduring resistance to blast [314]. Genetic mapping and molecular cloning of different blast resistance genes has provided a gamut of linked or gene-based markers for the efficient selection of resistance genes in breeding programs. Several blast resistance genes have been deployed in rice using different genomics-assisted approaches for achieving long-lasting resistance to blast (Table 5). Of the various *R* genes, the *Pi54* gene cloned from rice line Tetep in our laboratory [95] has been deployed more extensively, both globally and in India, in combination with other blast resistance genes for achieving durable resistance to blast (Table 6).

### 6.3. Transgenic Approach for Blast Management

The transgenic approach is one the important components in rice blast disease management. The initial cloning and characterization of the *R* genes was performed using the transgenic approach by expressing these genes in susceptible rice lines (Table 3). As discussed in details in the earlier section, 38 blast *R* were transformed into different rice lines. Although large-scale field release of these transgenic lines is not reported, the developed lines are a valuable resource for deployment whenever regulatory approval is given. Among the 38 cloned blast *R* genes, *Pi54* and its orthologs are widely studied using different methods through transgenics (Table 6).

### 6.4. Genome Editing of Immunity Regulators

Sequence-specific nucleases (SSNs), such as zinc finger nucleases (ZFNs), transcription activator-like effector nucleases (TALENs), and clustered regularly interspaced short palindromic repeats (CRISPR)/CRISPR-associated (Cas) 9 (CRISPR/Cas9) have recently proven to be extremely effective tools for plant genome editing [357]. Since it has become feasible to use the bacterial CRISPR/Cas mechanism in eukaryotes, which in itself is simple to design, fairly affordable, and multiplexing compliant [358], it has consequently superseded other approaches. CRISPR/Cas9 has proved to be the most effective SSN to date and has been used to alter the genomes of key crops including rice [359].

Susceptibility factor-encoding genes are often potential targets for genome editing as knockout of a single gene can significantly improve tolerance [360]. Müller and Munné-Bosch found that plant ethylene responsive factors (*ERF*) have a role in stress tolerance regulation [361]. Furthermore, RNAi silencing of rice *ERF922* in cultivar Zhonghua 17 improves resistance to *M. oryzae*, suggesting that this gene may function as a resistance negative regulator [362]. Consistent with these findings, the CRISPR/Cas9-targeted knockouts of *ERF* transcription factor have shown an improved resistance to rice blast [363]. The gene-edited mutants exhibited a reduced number of blast lesions following pathogen infection compared to wild-type plants at both the seedling and tillering stages. Furthermore, no significant differences were observed between mutant lines and the wild-type plants for different agronomic traits tested.

To investigate the functional relevance of exocyst subunit proteins in plants defense mechanisms, CRISPR/Cas9 was used to alter *OsSEC3A*, which is reported to be associated with rice defense responses. The two exons of the *OsSEC3A* were targeted with two sgRNAs [364]. Edited rice plants showed enhanced immunological response and enhanced resistance to the blast disease. Blast resistance can also be realized by fine-tuning the multifunctional genes involved in rice defense signaling. CRISPR/Cas9 multiplex genome editing system was used for targeted alteration of the thermosensitive male sterile 5 gene (*TMS5*), rice blast susceptibility gene *pi21*, and bacterial leaf blight susceptibility gene *xa13* [365]. Triple mutants (*tms5*/*pi21*/*xa13*) with homozygous frame-shift mutations in all three genes displayed thermosensitive genic male sterility with enhanced resistance to rice blast and bacterial blight.

Besides, CRISPR/Cas9 has also been used for functional validation of blast resistance genes. The *R* gene, *Pi-d2,* has been targeted for editing using the hAID*D-XTEN-Cas9n-NLS chimeric gene (dubbed *rBE5*) base editor to validate its role in resistance to *M. oryzae* [366]. Similarly, Zhao et al. [101] used CRISPR/Cas9 to confirm the function of the *Ptr*, a constitutively expressed resistance gene that imparts broad spectrum resistance to *M. oryzae*. Therefore, genome editing through CRISPR/Cas has more potential application in rice for developing varieties with enhanced blast resistance and also for the functional validation of potential defense response genes. Advanced genome-editing technologies such as base editing and prime editing could be used to install superior allelic variations precisely for developing blast resistance. In this direction, the editing of the susceptibility factors holds a great promise provided the targeted genes are chosen carefully to prevent fitness cost or yield reduction.

## 7. Conclusions and Future Perspective

Rice blast disease is a major threat to global rice production. Besides, *M. oryzae* is considered as the most potent potential biological weapon. Blast pathogen affects all parts of the rice plant from roots to panicles [4]. Since its detection dating back to 1637, constant efforts are being made to develop strategies for the effective management of rice blast disease. To date, more than 500 blast resistance QTLs have been reported, about 102 blast *R* genes have been mapped in rice, and 38 of these mapped genes are cloned and functionally characterized. The reported QTLs and *R* genes have been deployed in the genetic background of elite rice lines for resistance breeding using both conventional and genomics-assisted breeding approaches. The pathogen on the other hand displays an exceptional genomic plasticity that enables it to adapt to changes in the host, thus making it difficult for the rice breeders and researchers to rest on the present achievements to deal with the threat. There is always a chance of emergence of new virulent strains of a pathogen that can subvert the existing resistance responses [12]. Continued efforts to study the rice–*Magnaporthe* interaction, in order to understand the molecular mechanism of pathogenicity and resistance, are required to devise means to counteract the adaptability potential of the pathogen.

Though considerable success has been achieved in managing the disease through host resistance, the present changing climatic scenarios may alter this advantage in favor of the pathogen in the near future. As was reported in the case of the recent outbreak of wheat blast disease in Bangladesh, climate change is going to be a big challenge to manage various major as well as minor diseases [72]. The outbreak of wheat blast, reported to be an event of host-jump of *Magnaporthe* to wheat, highlights the potential host jump of *Magnaporthe* from other crops to rice, thereby risking everything we have achieved in rice blast management. The centuries of research and recent revelations in genomics indicate that we have almost exhausted our rice genetic resources for the identification of novel, potent resistance genes for tackling emerging strains of blast pathogen. Therefore, to this end, the recent developments in the field of genome editing, mainly in CRISPR-Cas systems, appears to hold on to the future challenges. Knock-out of single and multiple genes and induction of targeted genetic variation with conventional CRISPR-Cas tools and precise editing with base editors and prime editors empower us with the ability to decipher a great deal of host–pathogen interactions and improve rice plant for blast resistance. This is largely true because till now researchers have focused on the positive regulators of blast disease resistance in rice. There lies a plethora of rice negative regulators—many of which are yet to be identified—that have the potential to provide more stable and durable resistance.

## Figures and Tables

**Table 1:** List of *Magnaporthe oryzae* genome sequenced.

Strain	Genome Size (Mb)	N 50 Value	No. of Genes	% of Repeats in Genome	Reference
70-15	38.8	1.6 Mb	11,109	9.7	[11]
Ina168	38.0	28.4 Kb	NA	NA	[16]
P131	37.95	12.3 Kb	12,714	3.15	[13]
Y34	38.87	11.6 Kb	12,862	3.41	[13]
FJ81278	37.3	151.7 Kb	10,453	2.73	[14]
HN19311	37.1	147.4 Kb	10,256	2.83	[14]
98-06	42.1	88.6 Kb	14,019	9.3	[17]
B157	41	92.4 Kb	12,535	10.4	[15]
MG01	43	54.6 Kb	13,135	10.39	[15]
GFSI1-7-2	39.1	88.3 Kb	12,468	NA	[16]
Br48	40.7	97.5 Kb	12,671	NA	[16]
Br58	40.2	91.1 Kb	12,626	NA	[16]
Z2-1	39.5	64.1 Kb	12,383	NA	[16]
Dig41	41.3	24.3 Kb	11,457	NA	[16]
RML-29	42.2	10.4 Kb	12,746	11.78	[12]
RP-2421	44.85	35.35 Kb	12,957	12.28	[12]
2539	38.08	107 Kb	12,116	NA	[18]
RMg_Dl	42.42	524.2 Kb	10,555	NA	[19]
FR13	46.45	5.39 Mb	14,322	13.23	[20]
US71	45.61	2.81 Mb	14,348	13.23	[20]
CD156	43.39	5.53 Mb	14,304	6.21	[20]
BR32	41.85	5.09 Mb	NA	6.26	[20]
QJ08-2006	38.41	127.4 Kb	10,432	2.28	[21]
QJ10-10	38.28	105.1 Kb	10,418	2.22	[21]
QJ10-3001	38.40	133.1 Kb	10,401	2.28	[21]
RMg-Dl	34.82	45.894 Kb	12,747	NA	[22]
70-15	40.90	NA	12,991	11.1	[23]
FR13	42.40	0.104 Mb	14,384	1.56	[23]
GY11	39.00	0.226 Mb	14,781	1.00	[23]
PH14	40.00	0.757 Mb	13,816	1.16	[23]
TH12	40.10	0.716 Mb	14,026	1.46	[23]
TH16	39.10	0.939 Mb	13,571	1.60	[23]
US71	41.20	0.814 Mb	13,803	2.40	[23]
BR32	41.90	1.760 Mb	14,336	2.00	[23]
CD156	42.70	1.066 Mb	14,067	1.47	[23]
BR29	40.90	0.955 Mb	12,283	1.60	[23]
V86010	38.9	93.4 Kb	11,857	5.1	[24]
76_3	38.35	0.159 Mb	NA	NA	[25]
82_0835	40.07	0.136 Mb	NA	NA	[25]
90_4_1	39.92	0.151 Mb	NA	NA	[25]
BF17	39.72	0.138 Mb	NA	NA	[25]
BF32	40.18	0.120 Mb	NA	NA	[25]
BF48	40.01	0.144 Mb	NA	NA	[25]
BF5	40.96	0.122 Mb	NA	NA	[25]
BN0293	38.14	0.178 Mb	NA	NA	[25]
EG308	41.56	0.149 Mb	NA	NA	[25]
Glhn3	39.39	0.134 Mb	NA	NA	[25]
Glhn4	39.52	0.134 Mb	NA	NA	[25]
JUM1	40.50	0.127 Mb	NA	NA	[25]
KE002	40.25	0.147 Mb	NA	NA	[25]
KE016	40.27	0.156 Mb	NA	NA	[25]
KE017	40.10	0.141 Mb	NA	NA	[25]
KE019	39.37	0.176 Mb	NA	NA	[25]
KE021	40.07	0.152 Mb	NA	NA	[25]
KE029	41.03	0.154 Mb	NA	NA	[25]
KE041	39.00	0.146 Mb	NA	NA	[25]
KE210	38.85	15.86 Kb	NA	NA	[25]
KE255	39.83	0.119 Mb	NA	NA	[25]
KE332	41.00	35.0 Kb	NA	NA	[25]
KE415	39.45	33.91 Kb	NA	NA	[25]
KE443	40.88	36.41 Kb	NA	NA	[25]
KE473	40.61	37.27 Kb	NA	NA	[25]
KE491	40.05	34.50 Kb	NA	NA	[25]
NG0110	39.60	0.115 Mb	NA	NA	[25]
NG0135	39.85	0.109 Mb	NA	NA	[25]
NG0153	39.80	0.130 Mb	NA	NA	[25]
NGO104	38.94	0.128 Mb	NA	NA	[25]
TG004	39.96	0.125 Mb	NA	NA	[25]
TH3	37.30	22.62 Kb	NA	NA	[25]
TZ090	38.95	0.127 Mb	NA	NA	[25]
UG08	39.18	0.122 Mb	NA	NA	[25]
V0104	40.34	0.126 Mb	NA	NA	[25]
V0108	39.90	0.153 Mb	NA	NA	[25]
V0113	39.75	0.151 Mb	NA	NA	[25]

NA: Not available

**Table 2:** List of *Magnaporthe* Avr/effector genes cloned.

Avr Gene	Protein Size (aa)	Chromosome	Effector Type *	Cognate *R* Gene	Reference
*PWL1*	147	2	Glycine-rich	Unknown	[29]
*PWL2*	145	2	Glycine-rich	Unknown	[30]
*AVR1-CO39*	89	1	ToxB like	*Pi-CO39*	[31]
*AVR-Pita*	224	3	Zinc metalloprotease	*Pi-ta*	[32]
*ACE1*	4035	1	PKS/NRPS	*Pi33* (not cloned)	[33]
*AVR-Pia*	85	5 or 7 **	ToxB like	*Pia*	[16,34]
*AVR-Pii*	70	7	Unknown	*Pii*	[16]
*AVR-Pik/km/kp; (AVR-Pikh)*	113 (5 alleles)	1	ToxB like	*Pik/Pik-m/Pik-p, Pik-h*	[16,35]
*AvrPiz-t*	108	7	ToxB like	*Piz-t*	[36]
*AVR-Pi9*	91	7	Six cysteine	*Pi9*	[37]
*AVRPib*	75	3	Unknown	*Pib*	[38]
*AVR-Pi54*	153	4	ToxB like	*Pi54, Pi54rh, Pi54of*	[39]
*MoHTR1*	Unknown	Unknown	zinc-finger TF	Unknown	[28]
*MoHTR2*	Unknown	Unknown	zinc-finger TF	Unknown	[28]

PKS, polyketide synthase; NRPS, non-ribosomal peptide synthetase. * *Magnaporthe* Avrs and ToxB like (MAX)-effectors are classified based on protein 3-D models [38,39,40]. ** *AVR-Pia* is located on chromosome 5 (isolates Ina168 and Y93-165g-1; [34,41]) and chromosome 7 (isolate JS153; [42]).

**Table 5:** Resistance genes introgressed in rice for blast improvement program.

Target Gene	Recipient Parent	Chromosome	Marker Used	Reference
*Pi2*	C815S	6	RM527	[315]
*Pigm*	Kongyu 131, Longjing 26, Kenjiandao 6	6	M80362	[316]
*Pigm*	KT27S	6	G8900	[316]
*Pi9*	E32	6	Ins2-3	[317]
*Pi9*	R288	6	Clon2-1	[318]
*Pi9*	Q211S	6	Nbs21	[319]
*Pi40*	Osmancik-97, Halilbey	6	9871.T7E2b	[320]
*Pi1*	BPT5204	11	RM224	[321]
*Pi54*	BPT5204	11	Pi-54MAS	[322]
*Pi54*	R1, R2	11	RM224	[323]
*Pi54*	MTU1010	11	Pi54MAS/RM206	[324]
*Pi46/Pi-ta*	Hanghui 179	11, 12	RM224, YL155/YL87//YL155/87	[325]
*Pi1/Pi54/Pi-ta*	Mushk Budji	11, 12, 12	Pi54MAS, RM224, YL155/YL87//YL155/87	[323]
*Pib/Pik*	K6415	2, 11	NSb, K6415	[326]
*Pib/Pi54*	MR219	2, 11	RM208, RM206	[107]
*Pi1/Pi2*	GD-7S	6, 11	RM144, AP22	[327]
*Pi1/Pi2*	Pusa RH-10	6, 11	RM5926, AP5659-5	[326]
*Pi1/Pi2, Pi1/Pigm*	GZ63S, 97S, R084, R609	6, 11	RM224, ZJ58.7, AP22	[328]
*Pi2/Pi54*	PB1121	6, 11	AP5659-5, RM206	[329]
*Pi2/Pi54*	PRR78	6, 11	AP5930, RM206	[330]
*Piz-t/Pi54, Pi9/Pi54*	07GY31	6, 11	Z4794, Pikh-1	[331]
*Pi2/Pi1/Pi54*	Swarna-Sub1	6, 11	Pi54MAS, RM224, AP5659-5	[332]
*Pi1/Pi2/D12*	Jin 23B	6, 11, 12	RM144/RM224, PI2-4/HC28, RM277/RM309	[333]
*Pi9/Pi-ta*	Pusa Basmati 1	6, 12	AP5659-5, YL155/YL87//YL155/87	[334]
*Pi1/Pi2/Pi33*	Kuboyar	6, 8, 11	RM224, RM527, RM310	[335]
*Pi1/Pi2/Pi33*	ADT43	6, 8, 11	RM224, RM527, RM25	[336]
*Pi2/Pi54*	Sambha Mahsuri	6,11	Pi54MAS, AP5659-5	[337]

**Table 6:** Applications of *Pi54* gene in developing blast resistance rice.

Country	Approach	Applications	Cultivar Developed	Reference
China; Beijing	MAS-Gene pyramiding	*Pi9*, *Pizt*, *and Pi54* for blast resistance	NILs	[331]
China; Yangzhou	MAS-Gene pyramiding	Combination of major *R* genes including Pi54 for blast resistance	NILs	[338]
China; Wuhan	MAS-Gene pyramiding	*Pi54*, *Pi37*, *Pit*, *Pid3*, *Pigm*, *Pi36*, *Pi5, Pikm,* and *Pb1* for blast resistance	Improved Y58S, GuangZhan63S (GZ63), C815S and HD9802S	[339]
India (ICAR-IIRR)	MAS-Gene pyramiding	*Pi54* blast and *Xa21*, *xa13* blight resistance	MTU1010	[324]
India (ICAR-IARI)	MAS-Gene pyramiding	*Pi2*, *Pi54* blast and *xa13*, *Xa21* blight resistance	PB1121-NILs and PB6-NILs	[329]
India (ICAR-IIRR)	MAS-Gene pyramiding	*Pi54* blast and *Xa2* blight resistance	DRR17B	[340]
India (ICAR-IIRR)	MAS-Gene pyramiding	*Pi54* and *Pi2* blast resistance	Improved Samba Mahsuri	[337]
India (ICAR-IARI)	MAS-Gene pyramiding	*Pi54*, *Pi1*, *Pita*, *Pi2*, and *Pi9*	PB1 NILs	[314]
India (PJTSAU, Hyderabad)	MAS-Gene pyramiding	*Pi54* and *Pi1* for blast resistance	Tellahamsa	[341]
ICAR-IARI	MAS-Gene pyramiding	*Piz5* and *Pi54* blast resistance	Basmati restorer PRR78	[342]
ICAR-IIRR	MAS-Gene pyramiding	*Pi54* blast and *Xa2* blight resistance	IR58025B	[343]
ICAR-IARI	MAS-Gene pyramiding	*Pi54* blast, *xa13*, *Xa21* blight and QTL *qSBR11-1* ShB resistance	Improved Pusa Basmati 1	[344]
ICAR-IIRR	MAS	*Pi1, Pi2, Pi33,* and *Pi54* for blast resistance	ADT 43 NIL	[345]
ICAR-IIRR	MAS	*Pi1, Pi2,* and *Pi54*	16 introgressed lines	[346]
Universiti Putra, Malaysia	MABB	*Pi54 (Pi-kh)* and *Pi-b*	MR219	[327]
ICAR-IIRR	MABB	*Pi54* blast resistance	Swarna	[347]
ICAR-IIRR	MABB	*Pi54* introgression for blast resistance	Samba Mahsuri	[322]
ICAR-IIRR	MABB	*Pi2, Pi54, Xa21, xa13,* and *xa5*	Improved Samba Mahsuri	[348]
UAS & Tech, Kashmir	MABB	*Pi54, Pi1,* and *Pita*	Mushk Budji	[323]
ICAR-IIRR	MAS-Gene pyramiding	*PizPi1, Pi2,* and *Pi54*	Swarna-Sub1	[332]
China: Yangzhou	MAS-Gene pyramiding	*Pi1, Pi33,* and *Pi54, Piz*	15-pyramided lines	[349]
TNAU	MAS	*Pi54* introgression for blast resistance	Restorer lines	[350]
ICAR-IIRR	MAS-Gene pyramiding	*Pi54*, *Pi1, Xa21,* and *xa13*	Tellahamsa	[341]
ICAR-IARI	Allele mining	*Pi54* allele mining land races and wild rice	-	[351]
Switzerland (ETH Zurich)	Allele mining	*Pi54* mining from 885 Indian rice genotype	-	[279]
ICAR-NRCPB	Allele mining	*Pi54* mining from 92 rice lines	-	[293]
ICAR-IARI	Allele mining	*Pi54* mining from 100 rice germplasm	-	[352]
ICAR-NRCPB	Allele mining	*Pi54* mining from land races and wild rice	-	[170]
Tohoku University, Japan	Allele mining	*Pi54* evolution in the *Oryza* genus	-	[353]
China; Yancheng	Allele mining	Field resistance for blast *Pi-ta*, *Pigm,* and *Pi54* for blast disease	Rice accessions	[354]
Malaysia	Over-expression	Constitutive expression of *Pi54* homologue from rice line PH9	Transgenic- MR219	[355]
ICAR-NRCPB	Over-expression	*Pi54* orthologue from *O. officinalis*	Transgenic TP309	[89]
ICAR-NRCPB	Over-expression	*Pi54* orthologue from *O. rhizomatis*	Transgenic TP309	[171]
ICAR-NIPB	Over-expression	*Pi54*	TP309	[169]
China; Chengdu	Over-expression	*Pib, Pi25,* and *Pi54*	Kasalath, Zhenghan 10	[356]

## References

[1] Thirze H. (2016). Modelling Grain Surplus and Deficit in Cameroon for 2030. Master’s Thesis.

[2] Asibi A.E., Chai Q., Coulter J.A. (2019). Rice blast: A disease with implications for global food security. Agronomy.

[3] Samal P., Babu S. The shape of rice agriculture towards 2050. Proceedings of the Conference IAAE 30th International Conference of Agricultural Economists.

[4] Sharma T.R., Rai A.K., Gupta S.K., Vijayan J., Devanna B.N., Ray S. (2012). Rice blast management through host-plant resistance: Retrospect and prospects. Agric. Res..

[5] Kaundal R., Kapoor A.S., Raghava G.P. (2006). Machine learning techniques in disease forecasting: A case study on rice blast prediction. BMC Bioinform..

[6] Wang G.P., Lee S., Wang J., Ma L., Bianco T., Jia Y. (2014). Current advances on genetic resistance to rice blast disease. Rice-Germplasm Genet. Improv..

[7] Kato H. (2001). Rice blast disease. Pestic. Outlook.

[8] Gnanamanickam S.S. (2009). Rice and its importance to human life. Biological Control of Rice Diseases.

[9] Variar M. (2006). Pathogenic variation in *M. grisea* and breeding for blast resistance in India. JIRCAS Working Report No. 53.

[10] Wilson R.A., Talbot N.J. (2009). Under pressure: Investigating the biology of plant infection by *Magnaporthe oryzae*. Nat. Rev. Microbiol..

[11] Dean R.A., Talbot N.J., Ebbole D.J., Farman M.L., Mitchell T.K., Orbach M.J., Thon M., Kulkarni R., Xu J.R., Pan H. (2005). The genome sequence of the rice blast fungus *Magnaporthe grisea*. Nature.

[12] Singh P.K., Mahato A.K., Jain P., Rathour R., Sharma V., Sharma T.R. (2019). Comparative genomics reveals the high copy number variation of a retro transposon in different Magnaporthe isolates. Front. Microbiol..

[13] Xue M., Yang J., Li Z., Hu S., Yao N., Dean R.A., Zhao W., Shen M., Zhang H., Li C. (2012). Comparative analysis of the genomes of two field isolates of the rice blast fungus *Magnaporthe oryzae*. PLoS Genet..

[14] Chen C., Lian B., Hu J., Zhai H., Wang X., Venu R., Liu E., Wang Z., Chen M., Wang B. (2013). Genome comparison of two *Magnaporthe oryzae* field isolates reveals genome variations and potential virulence effectors. BMC Genom..

[15] Gowda M., Shirke M.D., Mahesh H.B., Chandarana P., Rajamani A., Chattoo B.B. (2015). Genome analysis of rice-blast fungus *Magnaporthe oryzae* field isolates from southern India. Genome Data.

[16] Yoshida K., Saitoh H., Fujisawa S., Kanzaki H., Matsumura H., Yoshida K., Tosa Y., Chuma I., Takano Y., Win J. (2009). Association genetics reveals three novel avirulence genes from the rice blast fungal pathogen *Magnaporthe oryzae*. Plant Cell.

[17] Dong Y., Li Y., Zhao M., Jing M., Liu X., Liu M., Guo X., Zhang X., Chen Y., Liu Y. (2015). Global genome and transcriptome analyses of Magnaporthe oryzae epidemic isolate 98-06 uncover novel effectors and pathogenicity-related genes, revealing gene gain and lose dynamics in genome evolution. PLoS Pathog..

[18] Chen M., Wang B., Lu G., Zhong Z., Wang Z. (2020). Genome sequence resource of *Magnaporthe oryzae* laboratory strain 2539. Mol. Plant Microbe Interact..

[19] Reddy B., Kumar A., Mehta S., Sheoran N., Chinnusamy V., Prakash G. (2021). Hybrid de novo genome-reassembly reveals new insights on pathways and pathogenicity determinants in rice blast pathogen *Magnaporthe oryzae* RMg_Dl. Sci. Rep..

[20] Langner T., Harant A., Gomez-Luciano L.B., Shrestha R.K., Malmgren A., Latorre S.M., Burbano H.A., Win J., Kamoun S. (2021). Genomic rearrangements generate hypervariable mini-chromosomes in host-specific isolates of the blast fungus. PLoS Genet..

[21] Chen K., Feng J., Chen S., Su J., Yang J., Wang C., Feng A., Chen B., Zhu X., Wang W. (2021). Comparative Analysis of the Genomes of Three Field Isolates of the Rice Blast Fungus Magnaporthe oryzae from Southern China. Agric. Sci..

[22] Kumar A., Sheoran N., Prakash G., Ghosh A., Chikara S.K., Rajashekara H., Singh U.D., Aggarwal R., Jain R.K. (2017). Genome sequence of a unique Magnaporthe oryzae RMg-Dl isolate from India that causes blast disease in diverse cereal crops, obtained using PacBio single-molecule and Illumina HiSeq2500 sequencing. Genome Announc..

[23] Chiapello H., Mallet L., Guerin C., Aguileta G., Amselem J., Kroj T., Ortega-Abboud E., Lebrun M.H., Henrissat B., Gendrault A. (2015). Deciphering genome content and evolutionary relationships of isolates from the fungus Magnaporthe oryzae attacking different host plants. Genome Biol. Evol..

[24] Zhu K.P., Bao J.D., Zhang L.H., Xue Y., Yuan L., Zhu M.H., Lin Q.Y., Ao Z., Zhen Z., Bo Z. (2017). Comparative analysis of the genome of the field isolate V86010 of the rice blast fungus Magnaporthe oryzae from Philippines. J. Integr. Agric..

[25] Were V.M., Mwongera D.T., Soanes D.M., Shrestha R.K., Ryder L., Foster A.J., Mutiga S.K., Rotich F., Win J., Langer T. (2021). Genome sequences of sixty Magnaporthe oryzae isolates from multiple host plant species. Zenedo.

[26] Mentlak T.A., Kombrink A., Shinya T., Ryder L.S., Otomo I., Saitoh H., Terauchi R., Nishizawa Y., Shibuya N., Tomma B.P.J. (2012). Effector-mediated suppression of chitin-triggered immunity by *Magnaporthe oryzae* is necessary for rice blast disease. Plant Cell.

[27] Yang C., Yu Y., Huang J., Meng F., Pang J., Zhao Q., Islam A., Xu N., Tian Y., Liu J. (2019). Binding of the *Magnaporthe oryzae* chitinase MoChia1 by a rice tetratricopeptide repeat protein allows free chitin to trigger immune responses. Plant Cell.

[28] Kim S., Kim C.Y., Park S.Y., Kim K.T., Jeon J., Chung H., Choi G., Kwon S., Choi J., Jeon J. (2020). Two nuclear effectors of the rice blast fungus modulate host immunity via transcriptional reprogramming. Nat. Commun..

[29] Kang S., Sweigard J.A., Valent B. (1995). The PWL host specificity gene family in the blast fungus Magnaporthe grisea. Mol. Plant Microbe Interact..

[30] Sweigard J.A., Carroll A.M., Kang S., Farrall L., Chumley F.G., Valent B. (1995). Identification, cloning, and characterization of PWL2, a gene for host species specificity in the rice blast fungus. Plant Cell.

[31] Farman M.L., Leong S.A. (1998). Chromosome walking to the AVR1-CO39 avirulence gene of Magnaporthe grisea: Discrepancy between the physical and genetic maps. Genetics.

[32] Orbach M.J., Farrall L., Sweigard J.A., Chumley F.G., Valent B. (2000). A telomeric avirulence gene determines efficacy for the rice blast resistance gene Pi-ta. Plant Cell.

[33] Böhnert H.U., Fudal I., Dioh W., Tharreau D., Notteghem J.L., Lebrun M.H. (2004). A putative polyketide synthase/peptide synthetase from Magnaporthe grisea signals pathogen attack to resistant rice. Plant Cell.

[34] Miki S., Matsui K., Kito H., Otsuka K., Ashizawa T., Yasuda N., Fukiya S., Sato J., Hirayae K., Fujita Y. (2009). Molecular cloning and characterization of the AVR-Pia locus from a Japanese field isolate of Magnaporthe oryzae. Mol. Plant Pathol..

[35] Wu W., Wang L., Zhang S., Li Z., Zhang Y., Lin F., Pan Q. (2014). Stepwise arms race between AvrPik and Pik alleles in the rice blast pathosystem. Mol. Plant-Microbe Interact..

[36] Li W., Wang B., Wu J., Lu G., Hu Y., Zhang X., Zhang Z., Zhao Q., Feng Q., Zhang H. (2009). The Magnaporthe oryzae avirulence gene AvrPiz-t encodes a predicted secreted protein that triggers the immunity in rice mediated by the blast resistance gene Piz-t. Mol. Plant-Microbe Interact..

[37] Wu J., Kou Y., Bao J., Li Y., Tang M., Zhu X., Ponaya A., Xiao G., Li J., Li C. (2015). Comparative genomics identifies the Magnaporthe oryzae avirulence effector AvrPi9 that triggers Pi9-mediated blast resistance in rice. New Phytol..

[38] Zhang S., Wang L., Wu W., He L., Yang X., Pan Q. (2015). Function and evolution of Magnaporthe oryzae avirulence gene AvrPib responding to the rice blast resistance gene Pib. Sci. Rep..

[39] Ray S., Singh P.K., Gupta D.K., Mahato A.K., Sarkar C., Rathour R., Singh N.K., Sharma T.R. (2016). Analysis of *Magnaporthe oryzae* genome reveals a fungal effector, which is able to induce resistance response in transgenic rice line containing resistance gene, Pi54. Front. Plant Sci..

[40] De Guillen K., Ortiz-Vallejo D., Gracy J., Fournier E., Kroj T., Padilla A. (2015). Structure analysis uncovers a highly diverse but structurally conserved effector family in phytopathogenic fungi. PLoS Pathog..

[41] Yasuda N., Tsujimoto Noguchi M., Fujita Y. (2006). Partial mapping of avirulence genes AVR-Pii and AVR-Pia in the rice blast fungus Magnaporthe oryzae. Can. J. Plant Pathol..

[42] Chen Q.H., Wang Y.C., Li A.N., Zhang Z.G., Zheng X.B. (2007). Molecular mapping of two cultivar-specific avirulence genes in the rice blast fungus Magnaporthe grisea. Mol. Genet. Genom..

[43] Wang B.H., Ebbole D.J., Wang Z.H. (2017). The arms race between *Magnaporthe oryzae* and rice: Diversity and interaction of Avr and R genes. J. Integr. Agric..

[44] Okuyama Y., Kanzaki H., Abe A., Yoshida K., Tamiru M., Saitoh H., Fujibe T., Matsumura H., Shenton M., Galam D.C. (2011). A multifaceted genomics approach allows the isolation of the rice Pia-blast resistance gene consisting of two adjacent NBS-LRR protein genes. Plant.

[45] Césari S., Kanzaki H., Fujiwara T., Bernoux M., Chalvon V., Kawano Y., Shimamoto K., Dodds P., Terauchi R., Kroj T. (2014). The NB-LRR proteins RGA 4 and RGA 5 interact functionally and physically to confer disease resistance. EMBO J..

[46] Zhao J.H., Zhang T., Liu Q.Y., Guo H.S. (2021). Trans-kingdom RNAs and their fates in recipient cells: Advances, utilization and perspectives. Plant Commun..

[47] Huang C.Y., Wang H., Hu P., Hamby R., Jin H. (2019). Small RNAs—Big players in plant-microbe interactions. Cell Host Microbe.

[48] Cai Q., Qiao L., Wang M., He B., Lin F.M., Palmquist J., Huang S.D., Jin H. (2018). Plants send small RNAs in extracellular vesicles to fungal pathogen to silence virulence genes. Science.

[49] LaMonte G., Philip N., Reardon J., Lacsina J.R., Majoros W., Chapman L., Thornburg C.D., Telen M.J., Ohler U., Nicchitta C.V. (2012). Translocation of sickle cell erythrocyte microRNAs into Plasmodium falciparum inhibits parasite translation and contributes to malaria resistance. Cell Host Microbe.

[50] Buck A.H., Coakley G., Simbari F., McSorley H.J., Quintana J.F., Le Bihan T., Kumar S., Abreu-Goodger C., Lear M., Harcus Y. (2014). Exosomes secreted by nematode parasites transfer small RNAs to mammalian cells and modulate innate immunity. Nat. Commun..

[51] Zhang T., Zhao Y.L., Zhao J.H., Wang S., Jin Y., Chen Z.Q., Fang Y.Y., Hua C.L., Ding S.W., Guo H.S. (2016). Cotton plants export microRNAs to inhibit virulence gene expression in a fungal pathogen. Nat. Plants.

[52] Weiberg A., Wang M., Lin F.M., Zhao H., Zhang Z., Kaloshian I., Huang H.D., Jin H. (2013). Fungal small RNAs suppress plant immunity by hijacking host RNA interference pathways. Science.

[53] Jiao J., Peng D. (2018). Wheat microRNA1023 suppresses invasion of *Fusarium graminearum* via targeting and silencing FGSG_03101. J. Plant Interact..

[54] Raman V., Simon S.A., Romag A., Demirci F., Mathioni S.M., Zhai J., Meyers B.C., Donofrio N.M. (2013). Physiological stressors and invasive plant infections alter the small RNA transcriptome of the rice blast fungus, *Magnaporthe oryzae*. BMC Genom..

[55] Raman V., Simon S.A., Demirci F., Nakano M., Meyers B.C., Donofrio N.M. (2017). Small RNA functions are required for growth and development of *Magnaporthe oryzae*. Mol. Plant-Microbe Interact..

[56] Nunes C.C., Gowda M., Sailsbery J., Xue M., Chen F., Brown D.E., Oh Y., Mitchell T.K., Dean R.A. (2011). Diverse and tissue-enriched small RNAs in the plant pathogenic fungus, *Magnaporthe oryzae*. BMC Genom..

[57] Zhang S., Xu J.R. (2014). Effectors and effector delivery in *Magnaporthe oryzae*. PLoS Pathog..

[58] Wang B., Sun Y., Song N., Zhao M., Liu R., Feng H., Wang X., Kang Z. (2017). *Puccinia striiformis* f. sp. tritici mi croRNA-like RNA 1 (Pst-milR1), an important pathogenicity factor of Pst, impairs wheat resistance to Pst by suppressing the wheat pathogenesis-related 2 gene. N. Phytol..

[59] Ji H.M., Mao H.Y., Li S.J., Feng T., Zhang Z.Y., Cheng L., Luo S.J., Borkovich K.A., Ouyang S.Q. (2021). Fol-milR1, a pathogenicity factor of Fusarium oxysporum, confers tomato wilt disease resistance by impairing host immune responses. N. Phytol..

[60] Zhang H., Liu S., Chang H., Zhan M., Qin Q.M., Zhang B., Li Z., Liu Y. (2019). Mining *Magnaporthe oryzae* sRNAs with potential Transboundary regulation of Rice genes associated with growth and defense through expression profile analysis of the pathogen-infected Rice. Front. Genet..

[61] Schuster M., Kahmann R. (2019). CRISPR-Cas9 genome editing approaches in filamentous fungi and oomycetes. Fungal Genet. Biol..

[62] Molla K.A., Karmakar S., Islam M.T., Islam M.T., Bhowmik P.K., Molla K.A. (2020). Wide horizons of CRISPR-cas-derived technologies for basic biology, agriculture, and medicine. CRISPR—Cas Methods.

[63] Foster A.J., Martin-Urdiroz M., Yan X., Wright H.S., Soanes D.M., Talbot N.J. (2018). CRISPR-Cas9 ribonucleoprotein-mediated co-editing and counterselection in the rice blast fungus. Sci. Rep..

[64] Liu R., Chen L., Jiang Y., Zhou Z., Zou G. (2015). Efficient genome editing in filamentous fungus *Trichoderma reesei* using the CRISPR/Cas9 system. Cell Discov..

[65] Pohl C., Kiel J.A., Driessen A.J., Bovenberg R.A., Nygard Y. (2016). CRISPR/Cas9 based genome editing of *Penicillium chrysogenum*. ACS Synth. Biol..

[66] Shi T.Q., Liu G.N., Ji R.Y., Shi K., Song P., Ren L.J., Huang H., Ji X.J. (2017). CRISPR/Cas9-based genome editing of the filamentous fungi: The state of the art. Appl. Microbiol. Biotechnol..

[67] Naduthodi M.I., Barbosa M.J., van der Oost J. (2018). Progress of CRISPR-Cas based genome editing in photosynthetic microbes. Biotechnol. J..

[68] Huang J., Cook D.E. (2022). CRISPR-Cas12a ribonucleoprotein-mediated gene editing in the plant pathogenic fungus *Magnaporthe oryzae*. STAR Protoc..

[69] Dean R., Van Kan J.A., Pretorius Z.A., Hammond-Kosack K.E., Di Pietro A., Spanu P.D., Rudd J.J., Dickman M., Kahmann R., Ellis J. (2012). The Top 10 fungal pathogens in molecular plant pathology. Mol. Plant Pathol..

[70] Malaker P.K., Barma N.C., Tiwary T.P., Collis W.J., Duveiller E.P., Singh K., Joshi A.K., Singh R.P., Braun H.J., Peterson G.L. (2016). First report of wheat blast caused by *Magnaporthe oryzae* pathotype triticum in Bangladesh. Plant Dis..

[71] Tembo B., Mulenga R.M., Sichilima S., M’siska K.K., Mwale M., Chikoti P.C., Singh P.K., He X., Pedley K.F., Peterson G.L. (2020). Detection and characterization of fungus (*Magnaporthe oryzae* pathotype *Triticum*) causing wheat blast disease on rain-fed grown wheat (*Triticum aestivum* L.) in Zambia. PLoS ONE.

[72] Devanna B.N., Sharma T.R. (2018). Wheat blast disease management: Cues from the advancements in molecular biology of rice-Magnaporthe pathosystem. J. Plant Biochem. Biotechnol..

[73] Kim K.H., Choi E.D. (2020). Retrospective study on the seasonal forecast-based disease intervention of the wheat blast outbreaks in Bangladesh. Front. Plant Sci..

[74] Islam M.T., Croll D., Gladieux P., Soanes D.M., Persoons A., Bhattacharjee P., Hossain M., Gupta D.R., Rahman M., Mahboob M.G. (2016). Emergence of wheat blast in Bangladesh was caused by a South American lineage of *Magnaporthe oryzae*. BMC Biol..

[75] Prabhu A.S., Filippi M.C., Castro N. (1992). Pathogenic variation among isolates of *Pyricularia oryzae* affecting rice, wheat, and grasses in Brazil. Int. J. Pest Manag..

[76] Urashima A.S., Igarashi S., Kato H. (1993). Host range, mating type, and fertility of *Pyricularia grisea* from wheat in Brazil. Plant Dis..

[77] Urashima A.S., Hashimoto Y., Don L.D., Kusaba M., Tosa Y., Nakayashiki H., Mayama S. (1999). Molecular analysis of the wheat blast population in Brazil with a homolog of retrotransposon MGR583. Jpn. J. Phytopathol..

[78] Urashima A.S., Galbieri R., Stabili A. (2005). DNA fingerprinting and sexual characterization revealed two distinct populations of *Magnaporthe grisea* in wheat blast from Brazil. Czech J. Genet. Plant Breed..

[79] Roy K.K., Reza M.M.A., Mustarin K.E., Malaker P.K., Barma N.C.D., He X., Singh P.K. (2021). First report of barley blast caused by *Magnaporthe oryzae* pathotype *Triticum* (MoT) in Bangladesh. J. Gen. Plant Pathol..

[80] Roy K.K., Rahman M.E., Reza M.A., Mustarin K.E., Malaker P.K., Barma N.C.D., Hossain I., He X., Singh P.K. (2021). First report of triticale blast caused by the fungus *Magnaporthe oryzae* pathotype *Triticum* in Bangladesh. Can. J. Plant Pathol..

[81] Eckardt N.A. (2000). Sequencing the rice genome. Plant Cell.

[82] Garris A.J., Tai T.H., Coburn J., Kresovich S., McCouch S. (2005). Genetic structure and diversity in *Oryza sativa* L.. Genetics.

[83] Song S., Tian D., Zhang Z., Hu S., Yu J. (2018). Rice genomics: Over the past two decades and into the future. Genom. Proteom. Bioinform..

[84] Du H., Yu Y., Ma Y., Gao Q., Cao Y., Chen Z., Ma B., Qi M., Li Y., Zhao X. (2017). Sequencing and de novo assembly of a near complete indica rice genome. Nat. Commun..

[85] Sharma T.R., Devanna B.N., Kiran K., Singh P.K., Arora K., Jain P., Tiwari I.M., Dubey H., Saklani B., Kumari M. (2018). Status and Prospects of Next generation Sequencing Technologies in Crop Plants. Curr. Issues Mol. Biol..

[86] GigaScience (2014). The 3000 Rice Genomes Project. GigaScience.

[87] Sun C., Hu Z., Zheng T., Lu K., Zhao Y., Wang W., Shi J., Wang C., Lu J., Zhang D. (2017). RPAN: Rice pan-genome browser for ∼3000 rice genomes. Nucleic Acids Res..

[88] Zhao Q., Feng Q., Lu H., Li Y., Wang A., Tian Q., Zhan Q., Lu Y., Zhang L., Huang T. (2018). Pan-genome analysis highlights the extent of genomic variation in cultivated and wild rice. Nat. Genet..

[89] Devanna N.B., Vijayan J., Sharma T.R. (2014). The blast resistance gene Pi54of cloned from *Oryza officinalis* interacts with Avr-Pi54 through its novel non-LRR domains. PLoS ONE.

[90] Thao N.P., Chen L., Nakashima A., Hara S.I., Umemura K., Takahashi A., Shirasu K., Kawasaki T., Shimamoto K. (2007). RAR1 and HSP90 form a complex with Rac/Rop GTPase and function in innate-immune responses in rice. Plant Cell.

[91] Li W., Chern M., Yin J., Wang J., Chen X. (2019). Recent advances in broad-spectrum resistance to the rice blast disease. Curr. Opin. Plant Biol..

[92] Faivre-Rampant O., Thomas J., Allègre M., Morel J.B., Tharreau D., Nottéghem J.L., Lebrun M.H., Schaffrath U., Piffanelli P. (2008). Characterization of the model system rice–Magnaporthe for the study of nonhost resistance in cereals. N. Phytol..

[93] Dai Y., Jia Y., Correll J., Wang X., Wang Y. (2010). Diversification and evolution of the avirulence gene AVR-Pita1 in field isolates of *Magnaporthe oryzae*. Fungal Genet. Biol..

[94] Liu G., Lu G., Zeng L., Wang G.L. (2002). Two broad-spectrum blast resistance genes, Pi9(t) and Pi2(t), are physically linked on rice chromosome 6. Mol. Genet. Genom..

[95] Sharma T.R., Madhav M.S., Singh B.K., Shanker P., Jana T.K., Dalal V., Pandit A., Singh A., Gaikwad K., Upreti H.C. (2005). High-resolution mapping, cloning and molecular characterization of the Pi-k h gene of rice, which confers resistance to *Magnaporthe grisea*. Mol. Genet. Genom..

[96] Fukuoka S., Saka N., Koga H., Ono K., Shimizu T., Ebana K., Hayashi N., Takahashi A., Hirochika H., Okuno K. (2009). Loss of function of a proline-containing protein confers durable disease resistance in rice. Science.

[97] Su J., Wang W., Han J., Chen S., Wang C., Zeng L., Feng A., Yang J., Zhou B., Zhu X. (2015). Functional divergence of duplicated genes results in a novel blast resistance gene Pi50 at the Pi2/9 locus. Theor. Appl. Genet..

[98] Chaipanya C., Telebanco-Yanoria M.J., Quime B., Longya A., Korinsak S., Korinsak S., Toojinda T., Vanavichit A., Jantasuriyarat C., Zhou B. (2017). Dissection of broad-spectrum resistance of the Thai rice variety Jao Hom Nin conferred by two resistance genes against rice blast. Rice.

[99] Dong L., Liu S., Xu P., Deng W., Li X., Tharreau D., Li J., Zhou J., Wang Q., Tao D. (2017). Fine mapping of Pi57(t) conferring broad spectrum resistance against *Magnaporthe oryzae* in introgression line IL-E1454 derived from *Oryza longistaminata*. PLoS ONE.

[100] Deng Y., Zhai K., Xie Z., Yang D., Zhu X., Liu J., Wang X., Qin P., Yang Y., Zhang G. (2017). Epigenetic regulation of antagonistic receptors confers rice blast resistance with yield balance. Science.

[101] Zhao H., Wang X., Jia Y., Minkenberg B., Wheatley M., Fan J., Jia M.H., Famoso A., Edwards J.D., Wamishe Y. (2018). The rice blast resistance gene Ptr encodes an atypical protein required for broad-spectrum disease resistance. Nat. Commun..

[102] Wang L., Zhao L., Zhang X., Zhang Q., Jia Y., Wang G., Li S., Tian D., Li W.H., Yang S. (2019). Large-scale identification and functional analysis of NLR genes in blast resistance in the Tetep rice genome sequence. Proc. Natl. Acad. Sci. USA.

[103] Kiyosawa S. (1970). Inheritance of a particular sensitivity of the rice variety, Sekiguchi Asahi, to pathogens and chemicals, and linkage relationship with blast resistance genes. Bull. Nat. Inst. Agric. Sci..

[104] Wang J., Qu B., Dou S., Li L., Yin D., Pang Z., Zhou Z., Tian M., Liu G., Xie Q. (2015). The E3 ligase OsPUB15 interacts with the receptor-like kinase PID2 and regulates plant cell death and innate immunity. BMC Plant Biol..

[105] Liu H., Dong S., Gu F., Liu W., Yang G., Huang M., Xiao W., Liu Y., Guo T., Wang H. (2017). NBS-LRR protein Pik-H4 interacts with OsBIHD1 to balance rice blast resistance and growth by coordinating ethylene-brassinosteroid pathway. Front. Plant Sci..

[106] Kang S.G., Lee K.E., Singh M., Kumar P., Matin M.N. (2021). Rice lesion mimic mutants (LMM): The current understanding of genetic mutations in the failure of ROS scavenging during lesion formation. Plants.

[107] Hayashi K., Yoshida H., Ashikawa I. (2006). Development of PCR-based allele-specific and InDel marker sets for nine rice blast resistance genes. Theor. Appl. Genet..

[108] Sallaud C., Lorieux M., Roumen E., Tharreau D., Berruyer R., Svestasrani P., Garsmeur O., Ghesquiere A., Notteghem J.-L. (2003). dentification of five new blast resistance genes in the highly blast-resistant rice variety IR64 using a QTL mapping strategy. Theor. Appl. Genet..

[109] Koizumi S. (2007). Durability of resistance to rice blast disease. JIRCAS Work. Rep..

[110] Barman S.R., Gowda M., Venu R.C., Chattoo B.B. (2004). Identification of a major blast resistance gene in the rice cultivar ‘Tetep’. Plant Breed..

[111] Nguyen T.T.T., Koizumi S., La T.N., Zenbayashi K.S., Ashizawa T., Yasuda N., Imazaki I., Miyasaka A. (2006). Pi35 (t), a new gene conferring partial resistance to leaf blast in the rice cultivar Hokkai 188. Theor. Appl. Genet..

[112] Lin F., Chen S., Que Z., Wang L., Liu X., Pan Q. (2007). The blast resistance gene Pi37 encodes a nucleotide binding site–leucine-rich repeat protein and is a member of a resistance gene cluster on rice chromosome 1. Genetics.

[113] Imbe T., Matsumoto S. (1985). Inheritance of resistance of rice varieties to the blast fungus strains virulent to the variety ‘‘Reiho’’. Jpn. J. Breed..

[114] Chen X.W., Li S.G., Xu J.C., Zhai W.X., Ling Z.Z., Ma B.T., Wang Y.P., Wang W.M., Cao G., Ma Y.Q. (2004). Identification of two blast resistance genes in a rice variety, Digu. J. Phytopathol..

[115] Zhu X., Yang Q., Yang J., Lei C., Wang J., Ling Z. (2004). Differentiation ability of monogenic lines to Magnaporthe grisea in indica rice. Acta Phytopathol Sin.

[116] Tabien R., Li Z., Paterson A., Marchetti M., Stansel J., Pinson S. (2002). Mapping QTLs for field resistance to the rice blast pathogen and evaluating their individual and combined utility in improved varieties. Theor. Appl. Genet..

[117] Lei C.L., Huang D.Y., Li W., Wang J.L., Liu Z.L., Wang X.T., Shi K., Cheng Z.J., Zhang X., Ling Z.Z. (2005). Molecular mapping of a blast resistance gene in an indica rice cultivar Yanxian No. 1. Rice Genet. Newsl..

[118] Wang Z.X., Yano M., Yamanouchi U., Iwamoto M., Monna L., Hayasaka H., Katayose Y., Sasaki T. (1999). The Pib gene for rice blast resistance belongs to the nucleotide binding and leucine-rich repeat class of plant disease resistance genes. Plant J..

[119] Wu K.S., Tanksley S.D. (1993). Abundance, polymorphism and genetic mapping of microsatellites in rice. Mol. Gen. Genet..

[120] Pan Q., Wang L., Ikehashi H., Tanisaka T. (1996). Identification of a new blast resistance gene in the indica rice cultivar Kasalath using Japanese differential cultivars and isozyme markers. Phytopathology.

[121] Utani D.W., Moeljopawiro S., Aswidinnoor H., Setiawan A., Hanarida I. (2008). Blast resistance genes in wild rice Oryza rufipogon and rice cultivar IR64 [online]. Indones. J. Agric..

[122] Tabien R.E., Pinson S.R.M., Marchetti M.A., Li Z., Park W.D., Paterson A.H., Stansel J.W. (1996). Blast resistance genes from Teqing and Lemont. Rice Genetics III: (In 2 Parts).

[123] Devi S.J.S.R., Singh K., Umakanth B., Vishalakshi B., Rao K.V.S., Suneel B., Sharma S.K., Kadambari G.K.M., Prasad M.S., Senguttvel P. (2020). Identification and characterization of a large effect QTL from *Oryza glumaepatula* revealed Pi68(t) as putative candidate gene for rice blast resistance. Rice.

[124] Pan Q.H., Tanisaka T., Ikehashi H. (1997). Studies on the genetics and breeding of blast resistance in rice VII. Gene analysis for the blast resistance of Indian native cultivar, Aus 373. Breed Sci..

[125] Ahn S.N., Kim Y.K., Hong H.C., Choi H.C., Moon H.P., Han S.S., Mccouch S.R. (1997). Mapping of genes conferring resistance to Korean isolates of rice blast fungus using DNA markers. Korean J. Breed..

[126] Goto I. (1988). Genetic Studieson Resistance of Rice Plant to Blast Fungus (VII) Blast Resistance Genes of Kuroka. Jpn. J. Phytopathol..

[127] Liu X., Yang Q., Lin F., Hua L., Wang C., Wang L., Pan Q. (2007). Identification and fine mapping of Pi39 (t), a major gene conferring the broad-spectrum resistance to *Magnaporthe oryzae*. Mol. Genet. Genom..

[128] Causse M.A., Fulton T.M., Cho Y.G., Ahn S.N., Chunwongse J., Wu K., Xiao J., Yu Z., Ronald P.C., Harrington S.E. (1994). Saturated molecular map of the rice genome based on an interspecific backcross population. Genetics.

[129] Naqvi N.I., Bonman J.M., Mackill D.J., Nelson R.J., Chattoo B.B. (1995). Identification of RAPD markers linked to a major blast resistance gene in rice. Mol. Breed..

[130] Wu J.L., Fan Y.Y., Li D.B., Zheng K.L., Leung H., Zhuang J.Y. (2005). Genetic control of rice blast resistance in the durably resistant cultivar Gumei 2 against multiple isolates. Theor. Appl. Genet..

[131] Jeung J.U., Kim B.R., Cho Y.C., Han S.S., Moon H.P., Lee Y.T., Jena K.K. (2007). A novel gene, Pi40 (t), linked to the DNA markers derived from NBS-LRR motifs confers broad spectrum of blast resistance in rice. Theor. Appl. Genet..

[132] Ahn S.N., Kim Y.K., Han S.S., Choi H.C., Moon H.P., McCouch S.R. (1996). Molecular mapping of a gene for resistance to a Korean isolate of rice blast. Rice Genet. Newsl..

[133] Qu S., Liu G., Zhou B., Bellizzi M., Zeng L., Dai L., Han B., Wang G.L. (2006). The broad-spectrum blast resistance gene Pi9 encodes a nucleotide-binding site–leucine-rich repeat protein and is a member of a multigene family in rice. Genetics.

[134] Mackill D.J., Bonman J.M. (1992). Inheritance of blast resistance in near-isogenic lines of rice. Phytopathology.

[135] Tabien R.E., Li Z., Paterson A.H., Marchetti M.A., Stansel J.W., Pinson S.R.M., Park W.D. (2000). Mapping of four major rice blast resistance genes from’Lemont’and’Teqing’and evaluation of their combinatorial effect for field resistance. Theor. Appl. Genet..

[136] Pan Q.H., Tanisaka T., Ikehashi H. (1996). Studies on the genetics and breeding of blast resistance in rice VI. Gene analysis for the blast resistance of two Yunnan native cultivars GA20 and GA25. Breed. Sci..

[137] Chen X., Shang J., Chen D., Lei C., Zou Y., Zhai W., Liu G., Xu J., Ling Z., Cao G. (2006). AB-lectin receptor kinase gene conferring rice blast resistance. Plant J..

[138] Deng Y., Zhu X., Shen Y., He Z. (2006). Genetic characterization and fine mapping of the blast resistance locus Pigm (t) tightly linked to Pi2 and Pi9 in a broad-spectrum resistant Chinese variety. Theor. Appl. Genet..

[139] Kinoshita T., Kiyosawa S. (1997). Some considerations on linkage relationships between Pii and Piz in the blast resistance of rice. Rice Genet. Newsl..

[140] Kwon S.W., Cho Y.C., Kim Y.G., Suh J.P., Jeung J.U., Roh J.H., Lee S.K., Jeon J.S., Yang S.J., Lee Y.T. (2008). Development of near-isogenic Japonica rice lines with enhanced resistance to Magnaporthe grisea. Mol. Cells.

[141] Ise K. (1991). Linkage analysis of some blast resistance gene in rice, *Oryza sativa* L.. Jpn. J. Breed..

[142] Chauhan R., Farman M.A.R.K., Zhang H.B., Leong S. (2002). Genetic and physical mapping of a rice blast resistance locus, Pi-CO39 (t), that corresponds to the avirulence gene AVR1-CO39 of Magnaporthe grisea. Mol. Genet. Genom..

[143] Wang G.L., Mackill D., Bonman J., McCouch S.R., Champoux M.C., Nelson R.J. (1994). RFLP mapping of genes conferring complete and partial resistance to blast in a durably resistant rice cultivar. Genetics.

[144] Zenbayashi K., Ashizawa T., Tani T., Koizumi S. (2002). Mapping of the QTL. Theor. Appl. Genet..

[145] Gowda M., Roy-Barman S., Chattoo B.B. (2006). Molecular mapping of a novel blast resistance gene Pi38 in rice using SSLP and AFLP markers. Plant Breed..

[146] Fujii K., Hayano-Saito Y., Shumiya A., Inoue M. (1995). Genetical mapping based on the RFLP analysis for the panicle blast resistance derived from a rice parental line St. No. 1. Breed. Sci..

[147] Fujii K., Hayano-Saito Y., Saito K., Sugiura N., Hayashi N., Tsuji T., Izawa T., Iwasaki M. (2000). Identification of a RFLP marker tightly linked to the panicle blast resistance gene *Pb1* in rice. Breed. Sci..

[148] Chen D.H., Inukai T., Mackill D.J., Ronald P.C., Nelson R.J. (1999). Molecular mapping of the blast resistance gene, Pi44 (t), in a line derived from a durably resistant rice cultivar. Theor. Appl. Genet..

[149] Hittalmani S., Parco A., Mew T.V., Zeigler R.S., Huang N. (2000). Fine mapping and DNA marker-assisted pyramiding of the three major genes for blast resistance in rice. Theor. Appl. Genet..

[150] Li L.Y., Wang L., Jing J.X., Li Z.Q., Lin F., Huang L.F., Pan Q.H. (2007). The Pik m gene, conferring stable resistance to isolates of Magnaporthe oryzae, was finely mapped in a crossover-cold region on rice chromosome 11. Mol. Breed..

[151] Hayasaka H., Miyao A., Yano M., Matsunaga K., Sasaki T. (1996). RFLP mapping of a rice blast resistance gene Pi-k. Breed Sci..

[152] Fjellstrom R., Conaway-Bormans C.A., McClung A.M., Marchetti M.A., Shank A.R., Park W.D. (2004). Development of DNA markers suitable for marker assisted selection of three Pi genes conferring resistance to multiple Pyricularia grisea pathotypes. Crop Sci..

[153] Goto I. (1970). Genetic studies on the resistance of rice plant to the blast fungus 1. Inheritance of resistance in crosses Sensho× H-79 and Imochi-shirazu× H-79. Jpn. J. Phytopathol..

[154] Shinoda H. (1971). Studies on the varietal resistance of rice to blast. 6. Linkage relationship of blast resistance genes. Annu. Rev. Chugoku Natl. Agric. Exp. Stn..

[155] Goto I. (1976). Genetic Studies on Resistance of Rice Plant to Blast Fungus II Difference in resistance to the blast disease between Fukunishiki and its parental cultivar, Zenith. Jpn. J. Phytopathol..

[156] Zhuang J.-Y., Ma W.-B., Wu J.-L., Chai R.-Y., Lu J., Fan Y.-Y., Jin M.-Z., Leung H., Zheng K.-L. (2002). Mapping of leaf and neck blast resistance genes with resistance gene analog, RAPD and RFLP in rice. Euphytica.

[157] Wu K.S., Martinez C., Lentini Z., Tohme J., Chumley F.G., Scolnik P.A., Valent B. (1996). Cloning a blast resistance gene by chromosome walking. Rice Genetics III: (In 2 Parts).

[158] McCouch S.R., Nelson R.J., Tohme J., Zeigler R.S. (1994). Mapping of blast resistance genes in rice. Rice Blast Disease.

[159] Inukai T., Nelson R.J., Zeigler R.S., Sarkarung S., Mackill D.J., Bonman J.M., Takamure I., Kinoshita T. (1996). Genetic analysis of blast resistance in tropical rice cultivars using near-isogenic lines. Rice Genetics III: (In 2 Parts).

[160] Nakamura S., Asakawa S., Ohmido N., Fukui K., Shimizu N., Kawasaki S. (1997). Construction of an 800-kb contig in the near-centromeric region of the rice blast resistance gene Pi-ta 2 using a highly representative rice BAC library. Mol. Gen. Genet..

[161] Iwata N. (1996). Registration of new gene symbols. Rice Genet. Newsl..

[162] Bryan G.T., Wu K.S., Farrall L., Jia Y., Hershey H.P., McAdams S.A., Faulk K.N., Donaldson G.K., Tarchini R., Valent B. (2000). A single amino acid difference distinguishes resistant and susceptible alleles of the rice blast resistance gene Pi-ta. Plant Cell.

[163] Liu W.G., Jin S.J., Zhu X.Y., Feng W.A.N.G., Li J.H., Liu Z.R., Liao Y.L., Zhu M.S., Huang H.J., Liu Y.B. (2008). Improving blast resistance of a thermo-sensitive genic male sterile rice line GD-8S by molecular marker-assisted selection. Rice Sci..

[164] Kaji R., Ogawa T. (1996). RFLP mapping of blast resistance gene Pi-km in rice. Breed Sci..

[165] Rai A.K., Kumar S.P., Gupta S.K., Gautam N., Singh N.K., Sharma T.R. (2011). Functional complementation of rice blast resistance gene Pi-k h (Pi54) conferring resistance to diverse strains of *Magnaporthe oryzae*. J. Plant Biochem. Biotechnol..

[166] Kumari M., Rai A.K., Devanna B.N., Singh P.K., Kapoor R., Rajashekara H., Prakash G., Sharma V. (2017). Co-transformation mediated stacking of blast resistance genes Pi54 and Pi54rh in rice provides broad spectrum resistance against *Magnaporthe oryzae*. Plant Cell Rep..

[167] Kumari M., Rai A.K., Devanna B.N., Singh P.K., Kapoor R., Rajashekara H., Prakash G., Sharma V., Sharma T.R. (2018). Stacking of blast resistance orthologue genes in susceptible indica rice line improves resistance against *Magnaporthe oryzae*. 3 Biotech.

[168] Arora K., Rai A.K., Devanna B.N., Kumari B., Sharma T.R. (2018). Functional validation of the Pi54 gene by knocking down its expression in a blast-resistant rice line using RNA interference and its effects on other traits. Funct. Plant Biol..

[169] Singh J., Gupta S.K., Devanna B.N., Singh S., Upadhyay A., Sharma T.R. (2020). Blast resistance gene Pi54 over-expressed in rice to understand its cellular and sub-cellular localization and response to different pathogens. Sci. Rep..

[170] Kumari A., Das A., Devanna B.N., Thakur S., Singh P.K., Singh N.K., Sharma T.R. (2013). Mining of rice blast resistance gene Pi54 shows effect of single nucleotide polymorphisms on phenotypic expression of the alleles. Eur. J. Plant Pathol..

[171] Das A., Soubam D., Singh P.K., Thakur S., Singh N.K., Sharma T.R. (2012). A novel blast resistance gene, Pi54rh cloned from wild species of rice, *Oryza rhizomatis* confers broad spectrum resistance to *Magnaporthe oryzae*. Funct. Integr. Genom..

[172] Sarkar C., Saklani B.K., Singh P.K., Asthana R.K., Sharma T.R. (2019). Variation in the LRR region of Pi54 protein alters its interaction with the AvrPi54 protein revealed by in silico analysis. PLoS ONE.

[173] Chen L., Hamada S., Fujiwara M., Zhu T., Thao N.P., Wong H.L., Krishna P., Ueda T., Kaku H., Shibuya N. (2010). The Hop/Sti1-Hsp90 chaperone complex facilitates the maturation and transport of a PAMP receptor in rice innate immunity. Cell Host Microbe.

[174] Nakashima A., Chen L., Thao N.P., Fujiwara M., Wong H.L., Kuwano M., Umemura K., Shirasu K., Kawasaki T., Shimamoto K. (2008). RACK1 functions in rice innate immunity by interacting with the Rac1 immune complex. Plant Cell.

[175] Lieberherr D., Thao N.P., Nakashima A., Umemura K., Kawasaki T., Shimamoto K. (2005). A sphingolipid elicitor-inducible mitogen-activated protein kinase is regulated by the small GTPase OsRac1 and heterotrimeric G-protein in rice. Plant Physiol..

[176] Arora K., Rai A.K., Devanna B.N., Dubey H., Narula A., Sharma T.R. (2021). Deciphering the role of microRNAs during Pi54 gene mediated *Magnaporthe oryzae* resistance response in rice. Physiol. Mol. Biol. Plants.

[177] Jones J.D., Dangl J.L. (2006). The plant immune system. Nature.

[178] Thomma B.P., Nürnberger T., Joosten M.H. (2011). Of PAMPs and effectors: The blurred PTI-ETI dichotomy. Plant Cell.

[179] Ngou B.P.M., Ahn H.K., Ding P., Jones J.D. (2021). Mutual potentiation of plant immunity by cell-surface and intracellular receptors. Nature.

[180] Yuan M., Ngou B.P.M., Ding P., Xin X.F. (2021). PTI-ETI crosstalk: An integrative view of plant immunity. Curr. Opin. Plant Biol..

[181] Waszczak C., Carmody M., Kangasjärvi J. (2018). Reactive oxygen species in plant signaling. Annu. Rev. Plant Biol..

[182] Gupta S.K., Rai A.K., Kanwar S.S., Chand D., Singh N.K., Sharma T.R. (2012). The single functional blast resistance gene Pi54 activates a complex defence mechanism in rice. J. Exp. Bot..

[183] Seo S., Mitsuhara I., Feng J., Iwai T., Hasegawa M., Ohashi Y. (2011). Cyanide, a coproduct of plant hormone ethylene biosynthesis, contributes to the resistance of rice to blast fungus. Plant Physiol..

[184] Norvienyeku J., Lin L., Waheed A., Chen X., Bao J., Aliyu S.R., Lin L., Shabbir A., Batool W., Zhong Z. (2021). Bayogenin 3-O-cellobioside confers non-cultivar-specific defence against the rice blast fungus *Pyricularia oryzae*. Plant Biotechnol. J..

[185] Dillon V.M., Overton J., Grayer R.J., Harborne J.B. (1997). Differences in phytoalexin response among rice cultivars of different resistance to blast. Phytochemistry.

[186] Koga J., Ogawa N., Yamauchi T., Kikuchi M., Ogasawara N., Shimura M. (1997). Functional moiety for the antifungal activity of phytocassane E, a diterpene phytoalexin from rice. Phytochemistry.

[187] Zhan C., Lei L., Liu Z., Zhou S., Yang C., Zhu X., Guo H., Zhang F., Peng M., Zhang M. (2020). Selection of a subspecies-specific diterpene gene cluster implicated in rice disease resistance. Nat. Plants.

[188] Yang C., Li W., Cao J., Meng F., Yu Y., Huang J., Jiang L., Liu M., Zhang Z., Chen X. (2017). Activation of ethylene signaling pathways enhances disease resistance by regulating ROS and phytoalexin production in rice. Plant J..

[189] Pieterse C.M.J., Van der Does D., Zamioudis C., Leon-Reyes A., Van Wees S.C.M. (2012). Hormonal modulation of plant immunity. Annu. Rev. Cell Dev. Biol..

[190] Silverman P., Seskar M., Kanter D., Schweizer P., Metraux J.P., Raskin I. (1995). Salicylic acid in rice (biosynthesis, conjugation, and possible role). Plant Physiol..

[191] Zhang X., Bao Y., Shan D., Wang Z., Song X., Wang Z., Wang J., He L., Wu L., Zhang Z. (2018). *Magnaporthe oryzae* induces the expression of a MicroRNA to suppress the immune response in rice. Plant Physiol..

[192] Patkar R.N., Naqvi N.I. (2017). Fungal manipulation of hormone-regulated plant defense. PLoS Pathog..

[193] Jiang C.J., Liu X.L., Liu X.Q., Zhang H., Yu Y.J., Liang Z.W. (2017). Stunted growth caused by blast disease in rice seedlings is associated with changes in phytohormone signaling pathways. Front. Plant Sci..

[194] Kyndt T., Zemene H.Y., Haeck A., Singh R., De Vleesschauwer D., Denil S., De Meyer T., Höfte M., Demeestere K., Gheysen G. (2017). Below-ground attack by the root knot nematode *Meloidogyne graminicola* predisposes rice to blast disease. Mol. Plant-Microbe Interact..

[195] Jiang C.J., Shimono M., Sugano S., Kojima M., Liu X., Inoue H., Sakakibara H. (2013). and Takatsuji, H.; Cytokinins act synergistically with salicylic acid to activate defense gene expression in rice. Mol. Plant-Microbe Interact..

[196] Zhang S., Deng Y.Z., Zhang L.H. (2018). Phytohormones: The chemical language in *Magnaporthe oryzae*-rice pathosystem. Mycology.

[197] Yazawa K., Jiang C.J., Kojima M., Sakakibara H., Takatsuji H. (2012). Reduction of abscisic acid levels or inhibition of abscisic acid signaling in rice during the early phase of *Magnaporthe oryzae* infection decreases its susceptibility to the fungus. Physiol. Mol. Plant Pathol..

[198] Sharma T.R., Das A., Thakur S., Devanna B.N., Singh P.K., Jain P., Vijayan J., Kumar S. (2016). Oscillating transcriptome during rice-Magnaporthe interaction. Curr. Issues Mol. Biol..

[199] Jain P., Dubey H., Singh P.K., Solanke A.U., Singh A.K., Sharma T.R. (2019). Deciphering signalling network in broad spectrum Near Isogenic Lines of rice resistant to *Magnaporthe oryzae*. Sci. Rep..

[200] Jain P., Singh P.K., Kapoor R., Khanna A., Solanke A.U., Krishnan S.G., Singh A.K., Sharma V., Sharma T.R. (2017). Understanding host-pathogen interactions with expression profiling of NILs carrying rice-blast resistance Pi9 gene. Front. Plant Sci..

[201] Arlorio M., Ludwig A., Boller T., Bonfante P. (1992). Inhibition of fungal growth by plant chitinases andβ-1,3-glucanases. Protoplasma.

[202] Bennett R.N., Wallsgrove R.M. (1994). Secondary metabolites in plant defence mechanisms. N. Phytol..

[203] Wang Y., Kwon S.J., Wu J., Choi J., Lee Y.H., Agrawal G.K., Tamogami S., Rakwal R., Park S.R., Kim B.G. (2014). Transcriptome analysis of early responsive genes in rice during *Magnaporthe oryzae* infection. Plant Pathol. J..

[204] Wally O., Punja Z.K. (2010). Enhanced disease resistance in transgenic carrot (*Daucus carota* L.) plants over-expressing a rice cationic peroxidase. Planta.

[205] Mittler R., Vanderauwera S., Gollery M., Van Breusegem F. (2004). Reactive oxygen gene network of plants. Trends Plant Sci..

[206] Zhang Y., Zhao J., Li Y., Yuan Z., He H., Yang H., Qu H., Ma C., Qu S. (2016). Transcriptome analysis highlights defense and signaling pathways mediated by rice pi21 gene with partial resistance to *Magnaporthe oryzae*. Front. Plant Sci..

[207] Delteil A., Gobbato E., Cayrol B., Estevan J., Michel-Romiti C., Dievart A., Kroj T., Morel J.-B. (2016). Several wall-associated kinases participate positively and negatively in basal defense against rice blast fungus. BMC Plant Biol..

[208] Albrecht C., Boutrot F., Segonzac C., Schwessinger B., Gimenez-Ibanez S., Chinchilla D., Rathjen J.P., de Vries S.C., Zipfel C. (2012). Brassinosteroids inhibit pathogen-associated molecular pattern–triggered immune signaling independent of the receptor kinase BAK1. Proc. Natl. Acad. Sci. USA.

[209] Liu W., Liu J., Triplett L., Leach J.E., Wang G.-L. (2014). Novel insights into rice innate immunity against bacterial and fungal pathogens. Annu. Rev. Phytopathol..

[210] Chujo T., Takai R., Akimoto-Tomiyama C., Ando S., Minami E., Nagamura Y., Kaku H., Shibuya N., Yasuda M., Nakashita H. (2007). Involvement of the elicitor-induced gene OsWRKY53 in the expression of defense-related genes in rice. Biochim. Biophys. Acta—Gene Struct. Expr..

[211] Vijayan J., Jain S., Jain N., Devanna B.N., Rathour R., Variar M., Prashanthi S.K., Singh A.K., Singh U.D., Singh N.K. (2013). Identification of differentially expressed genes in rice during its early phases of interaction with *Magnaporthe oryzae*. Indian J. Genet..

[212] Peng Y., Bartley L.E., Canlas P., Ronald P.C. (2010). OsWRKY IIa transcription factors modulate rice innate immunity. Rice.

[213] Bagnaresi P., Biselli C., Orrù L., Urso S., Crispino L., Abbruscato P., Piffanelli P., Lupotto E., Cattivelli L., Valè G. (2012). Comparative transcriptome profiling of the early response to *Magnaporthe oryzae* in durable resistant vs susceptible rice (*Oryza sativa* L.) genotypes. PLoS ONE.

[214] Wei T., Ou B., Li J., Zhao Y., Guo D., Zhu Y., Chen Z., Gu H., Li C., Qin G. (2013). Transcriptional profiling of rice early response to *Magnaporthe oryzae* identified *OsWRKYs* as important regulators in rice blast resistance. PLoS ONE.

[215] Dodds P.N., Rathjen J.P. (2010). Plant immunity: Towards an integrated view of plant–pathogen interactions. Nat. Rev. Genet..

[216] Mei C., Qi M., Sheng G., Yang Y. (2006). Inducible overexpression of a rice allene oxide synthase gene increases the endogenous jasmonic acid level, PR gene expression, and host resistance to fungal infection. Mol. Plant-Microbe Interact..

[217] Yamada S., Kano A., Tamaoki D., Miyamoto A., Shishido H., Miyoshi S., Taniguchi S., Akimitsu K., Gomi K. (2012). Involvement of OsJAZ8 in jasmonate-induced resistance to bacterial blight in rice. Plant Cell Physiol..

[218] Umemura K., Ogawa N., Shimura M., Koga J., Usami H., Kono T. (2003). Possible role of phytocassane, rice phytoalexin, in disease resistance of rice against the blast fungus *Magnaporthe grisea*. Biosci. Biotechnol. Biochem..

[219] Okada K. (2011). The biosynthesis of isoprenoids and the mechanisms regulating it in plants. Biosci. Biotechnol. Biochem..

[220] Jwa N.-S., Agrawal G.K., Tamogami S., Yonekura M., Han O., Iwahashi H., Rakwal R. (2006). Role of defense/stress-related marker genes, proteins and secondary metabolites in defining rice self-defense mechanisms. Plant Physiol. Biochem..

[221] Hasegawa M., Mitsuhara I., Seo S., Imai T., Koga J., Okada K., Yamane H., Ohashi Y. (2010). Phytoalexin accumulation in the interaction between rice and the blast fungus. Mol. Plant-Microbe Interact..

[222] Yamane H. (2013). Biosynthesis of phytoalexins and regulatory mechanisms of it in rice. Biosci. Biotechnol. Biochem..

[223] Tang J., Chu C. (2017). MicroRNAs in crop improvement: Fine-tuners for complex traits. Nat. Plants.

[224] Yao S., Yang Z., Yang R., Huang Y., Guo G., Kong X., Lan Y., Zhou T., Wang H., Wang W. (2019). Transcriptional regulation of miR528 by OsSPL9 orchestrates antiviral response in rice. Mol. Plant.

[225] Baldrich P., Campo S., Wu M.T., Liu T.T., Hsing Y.I.C., Segundo B.S. (2015). MicroRNA-mediated regulation of gene expression in the response of rice plants to fungal elicitors. RNA Biol..

[226] Zhang D., Liu M., Tang M., Dong B., Wu D., Zhang Z., Zhou B. (2015). Repression of microRNA biogenesis by silencing of OsDCL1 activates the basal resistance to *Magnaporthe oryzae* in rice. Plant Sci..

[227] Li Y., Jeyakumar J.M.J., Feng Q., Zhao Z.-X., Fan J., Khaskheli M.I., Wang W.-M. (2019). The roles of rice microRNAs in rice-*Magnaporthe oryzae* interaction. Phytopathol. Res..

[228] Singh P.K., Ray S., Thakur S., Rathour R., Sharma V., Sharma T.R. (2018). Co-evolutionary interactions between host resistance and pathogen avirulence genes in rice-*Magnaporthe oryzae* pathosystem. Fungal Genet. Biol..

[229] Flor H.H. (1971). Current status of the gene-for-gene concept. Annu. Rev. Phytopathol..

[230] Sharma T.R., Das A., Thakur S., Jalali B.L. (2014). Recent Understanding on Structure, Function and Evolution of Plant Disease Resistance Genes. Proc. Indian Natl. Sci. Acad..

[231] Sharma T.R., Das A., Kumar S.P., Lodha M.L. (2009). Resistance Gene Analogues as a Tool for Rapid Identification and Cloning of Disease Resistance genes in Plants. J. Plant Biochem. Biotechnol..

[232] Pruitt R.N., Schwessinger B., Joe A., Thomas N., Liu F., Albert M., Robinson M.R., Chan L.J.G., Luu D.D., Chen H. (2015). The rice immune receptor XA21 recognizes a tyrosine-sulfated protein from a Gram-negative bacterium. Sci. Adv..

[233] Van der Biezen E.A., Jones J.D.G. (1998). Plant disease- resistance proteins and the gene-for-gene concept. Trends Plant Sci..

[234] Dangl J.L., Jones J.D.G. (2001). Plant pathogens and integrated defence responses to infection. Nature.

[235] Ashikawa I., Hayashi N., Yamane H., Kanamori H., Wu J., Matsumoto T., Ono K., Yano M. (2008). Two adjacent nucleotide-binding site–leucine-rich repeat class genes are required to confer Pikm-specific rice blast resistance. Genetics.

[236] Lee S.K., Song M.Y., Seo Y.S., Kim H.K., Ko S., Cao P.J., Suh J.P., Yi G., Roh J.H., Lee S. (2009). Rice Pi5-mediated resistance to *Magnaporthe oryzae* requires the presence of two coiled-coil–nucleotide-binding–leucine-rich repeat genes. Genetics.

[237] Yuan B., Zhai C., Wang W., Zeng X., Xu X., Hu H., Lin F., Wang L., Pan Q. (2011). The Pik-p resistance to *Magnaporthe oryzae* in rice is mediated by a pair of closely linked CC-NBS-LRR genes. Theor. Appl. Genet..

[238] Zhai C., Zhang Y., Yao N., Lin F., Liu Z., Dong Z., Wang L., Pan Q. (2014). Function and interaction of the coupled genes responsible for Pik-h encoded rice blast resistance. PLoS ONE.

[239] Paulus J.K., van der Hoorn R.A.L. (2018). Tricked or trapped—Two decoy mechanisms in host–pathogen interactions. PLoS Pathol..

[240] Kim S.H., Qi D., Ashfield T., Helm M., Innes R.W. (2016). Using decoys to expand the recognition specificity of a plant disease resistance protein. Science.

[241] Kroj T., Chanclud E., Michel-Romiti C., Grand X., Morel J.B. (2016). Integration of decoy domains derived from protein targets of pathogen effectors into plant immune receptors is widespread. N. Phytol..

[242] Ortiz D., de Guillen K., Cesari S., Chalvon V., Gracy J., Padilla A., Kroj T. (2017). Recognition of the *Magnaporthe oryzae* effector AVR-Pia by the decoy domain of the rice NLR immune receptor RGA5. Plant Cell.

[243] Kearsey M.J., Pooni H.S. (1998). The Genetical Analysis of Quantitative Traits.

[244] Lopez-Gerena J. (2006). Mapping QTL Controlling Durable Resistance to Rice Blast in the Cultivar Oryzica Llanos 5. Ph.D. Thesis.

[245] Bonman J.M., Mackill D. (1988). Durable resistance to rice blast disease. Oryza.

[246] Zenbayashi-Sawata K., Ashizawa T., Koizumi S. (2005). Pi34-AVRPi34: A new gene-for-gene interaction for partial resistance in rice to blast caused by *Magnaporthe grisea*. J. Genet. Plant Pathol..

[247] Yunoki T., Ezuka A., Morinaka Y. (1970). Sakurai, Studies on the varietal resistance to rice blast. 4. Variation of field resistance due to fungus strains. Bull. Chugoku Agric. Exp. Stn. Ser. A.

[248] Fukuoka S., Okuno K. (2001). QTL analysis and mapping of pi21, a recessive gene for field resistance to rice blast in Japanese upland rice. Theor. Appl. Genet..

[249] Latif M., Yusop M.R., Rahman M.M., Talukdar M.B. (2011). Microsatellite and minisatellite markers based DNA fingerprinting and genetic diversity of blast and ufra resistant genotypes. Comptes Rendus Biol..

[250] Kou Y., Wang S. (2010). Broad-spectrum and durability: Understanding of quantitative disease resistance. Curr. Opin. Plant Biol..

[251] Wang J., Liu X., Zhang A., Ren Y., Wu F., Wang G., Xu Y., Lei C., Zhu S., Pan T. (2019). A cyclic nucleotide--gated channel mediates cytoplasmic calcium elevation and disease resistance in rice. Cell Res..

[252] Hayashi N., Inoue H., Kato T., Funao T., Shirota M., Shimizu T., Kanamori H., Yamane H., Hayano-Saito Y., Matsumoto T. (2010). Durable panicle blast-resistance gene *Pb1* encodes an atypical CC-NBS-LRR protein and was generated by acquiring a promoter through local genome duplication. Plant J..

[253] Chen J., Shi Y., Liu W., Chai R., Fu Y., Zhuang J., Wu J. (2011). A Pid3 allele from rice cultivar Gumei 2 confers resistance to *Magnaporthe oryzae*. J. Genet. Genom..

[254] Ma J., Lei C., Xu X., Hao K., Wang J., Cheng Z., Ma X., Zhou K., Zhang X., Guo X. (2015). Pi64, encoding a novel CC-NBS-LRR protein, confers resistance to leaf and neck blast in rice. Mol. Plant Microbe Interact..

[255] Cao N., Chen Y., Ji Z., Zeng Y., Yang C., Liang Y. (2019). Recent progress in molecular mechanism of rice blast resistance. Chin. J. Rice Sci..

[256] Li Y., Wu C., Xing Y., Chen H., He Y. (2008). Dynamic QTL analysis for rice blast resistance under natural infection conditions. Aust. J. Crop Sci..

[257] Takahashi A., Hayashi N., Miyao A., Hirochika H. (2010). Unique features of the rice blast resistance Pish locus revealed by large scale retrotransposon-tagging. BMC Plant Biol..

[258] Hayashi K., Yoshida H. (2009). Refunctionalization of the ancient rice blast disease resistance gene Pit by the recruitment of a retrotransposon as a promoter. Plant J..

[259] Wang Q., Li Y., Ishikawa K., Kosami K.I., Uno K., Nagawa S., Tan L., Du J., Shimamoto K., Kawano Y. (2018). Resistance protein Pit interacts with the GEF OsSPK1 to activate OsRac1 and trigger rice immunity. Proc. Natl. Acad. Sci..

[260] Xu X., Hayashi N., Wang C.-T., Fukuoka S., Kawasaki S., Takatsuji H., Jiang C.-J. (2014). Rice blast resistance gene *Pikahei-1*(t), a member of a resistance gene cluster on chromosome 4, encodes a nucleotide-binding site and leucine-rich repeat protein. Mol. Breed..

[261] Liu M.H., Kang H., Xu Y., Peng Y., Wang D., Gao L., Wang X., Ning Y., Wu J., Liu W. (2020). Genome-wide association study identifies an NLR gene that confers partial resistance to Magnaporthe oryzae in rice. Plant Biotechnol. J..

[262] Zhou B., Qu S., Liu G., Dolan M., Sakai H., Lu G., Bellizzi M., Wang G.L. (2006). The eight amino-acid differences within three leucine-rich repeats between Pi2 and Piz-t resistance proteins determine the resistance specificity to Magnaporthe grisea. Mol. Plant-Microbe Interact..

[263] Xie Z., Yan B., Shou J., Tang J., Wang X., Zhai K., Liu J., Li Q., Luo M., Deng Y. (2019). A nucleotide-binding site-leucine-rich repeat receptor pair confers broad-spectrum disease resistance through physical association in rice. Philos. Trans. R. Soc. B.

[264] Zhai K., Deng Y., Liang D., Tang J., Liu J., Yan B., Yin X., Lin H., Chen F., Yang D. (2019). RRM transcription factors interact with NLRs and regulate broad-spectrum blast resistance in rice. Mol. Cell.

[265] Shang J., Tao Y., Chen X., Zou Y., Lei C., Wang J., Li X., Zhao X., Zhang M., Lu Z. (2009). Identification of a new rice blast resistance gene, *Pid3*, by genome-wide comparison of paired nucleotide-binding site-leucine-rich repeat genes and their pseudogene alleles between the two sequenced rice genomes. Genetics.

[266] Zhou Z., Pang Z., Zhao S., Zhang L., Lv Q., Yin D., Li D., Liu X., Zhao X., Li X. (2019). Importance of OsRac1 and RAI1 in signalling of nucleotide-binding site leucine-rich repeat protein-mediated resistance to rice blast disease. New Phytol..

[267] Lv Q., Xu X., Shang J., Jiang G., Pang Z., Zhou Z., Wang J., Liu Y., Li T., Li X. (2013). Functional analysis of Pid3-A4, an ortholog of rice blast resistance gene Pid3 revealed by allele mining in common wild rice. Phytopathology.

[268] Liu X., Lin F., Wang L., Pan Q. (2007). The in silico map-based cloning of Pi36, a rice coiled-coil–nucleotide-binding site–leucine-rich repeat gene that confers race-specific resistance to the blast fungus. Genetics.

[269] Takagi H., Uemura A., Yaegashi H., Tamiru M., Abe A., Mitsuoka C., Utsushi H., Natsume S., Kanzaki H., Matsumura H. (2013). MutMap-Gap: Whole-genome resequencing of mutant F 2 progeny bulk combined with de novo assembly of gap regions identifies the rice blast resistance gene Pii. New Phytol..

[270] Liu Y., Liu B., Zhu X., Yang J., Bordeos A., Wang G., Leach J.E., Leung H. (2013). Fine-mapping and molecular marker development for Pi56 (t), a NBS-LRR gene conferring broad-spectrum resistance to Magnaporthe oryzae in rice. Theor. Appl. Genet..

[271] Inoue H., Hayashi N., Matsushita A., Xinqiong L., Nakayama A., Sugano S., Jiang C.J., Takatsuji H. (2013). Blast resistance of CC-NB-LRR protein Pb1 is mediated by WRKY45 through protein–protein interaction. Proc. Natl. Acad. Sci. USA.

[272] Zhai C., Lin F., Dong Z., He X., Yuan B., Zeng X., Wang L., Pan Q. (2011). The isolation and characterization of Pik, a rice blast resistance gene which emerged after rice domestication. New Phytol..

[273] Chen J., Peng P., Tian J., He Y., Zhang L., Liu Z., Yin D., Zhang Z. (2015). Pike, a rice blast resistance allele consisting of two adjacent NBS–LRR genes, was identified as a novel allele at the Pik locus. Mol. Breed..

[274] Hua L., Wu J., Chen C., Wu W., He X., Lin F., Wang L., Ashikawa I., Matsumoto T., Wang L. (2012). The isolation of Pi1, an allele at the Pik locus which confers broad spectrum resistance to rice blast. Theoretical and Applied Genetics.

[275] Sharma T.R., Rai A.K., Gupta S.K., Singh N.K. (2010). Broad-spectrum blast resistance gene pi-k h cloned from rice line Tetep designated as Pi54. J. Plant Biochem. Biotechnol..

[276] Chen L., Shiotani K., Togashi T., Miki D., Aoyama M., Wong H.L., Kawasaki T., Shimamoto K. (2010). Analysis of the Rac/Rop small GTPase family in rice: Expression, subcellular localization and role in disease resistance. Plant Cell Physiol..

[277] Ribot C., Cesari S., Abidi I., Chalvon V., Bournaud C., Vallet J., Lebrun M.H., Morel J.B., Kroj T. (2013). The M agnaporthe oryzae effector AVR 1–CO 39 is translocated into rice cells independently of a fungal-derived machinery. Plant J..

[278] Wang L., Ma Z., Kang H., Gu S., Mukhina Z., Wang C., Wang H., Bai Y., Sui G., Zheng W. (2022). Cloning and functional analysis of the novel rice blast resistance gene Pi65 in japonica rice. Theor. Appl. Genet..

[279] Vasudevan K., Gruissem W., Bhullar N.K. (2015). Identification of novel alleles of the rice blast resistance gene Pi54. Sci. Rep..

[280] Leung H., Raghavan C., Zhou B., Oliva R., Choi I.R., Lacorte V., Jubay M.L., Cruz C.V., Gregorio G., Singh R.K. (2015). Allele mining and enhanced genetic recombination for rice breeding. Rice.

[281] Till B.J., Reynolds S.H., Greene E.A., Codomo C.A., Enns L.C., Johnson J.E., Burtner C., Odden A.R., Young K., Taylor N.E. (2003). Large-scale discovery of induced point mutations with high-throughput TILLING. Genome Res..

[282] Kumar G.R., Sakthivel K., Sundaram R.M., Neeraja C.N., Balachandran S.M., Rani N.S., Viraktamath B.C., Madhav M.S. (2010). Allele mining in crops: Prospects and potentials. Biotechnol. Adv..

[283] Rawal H.C., Mithra S.V.A., Arora K., Kumar V., Goel N., Mishra D.C., Chaturvedi K.K., Rai A., Devi S.V., Sharma T.R. (2018). Genome-wide analysis in wild and cultivated Oryza species reveals abundance of NBS genes in progenitors of cultivated rice. Plant Mol. Biol. Report..

[284] Yang M.-Z., Cheng Z.-Q., Chen S.-N., Qian J., Xu L.-L., Huang X.-Q. (2007). A rice blast resistance genetic resource from wild rice in Yunnan, China, J. Plant Physiol. Mol. Biol..

[285] Geng X.S., Yang M.Z., Huang X.Q., Cheng Z.Q., Fu J., Sun T., Li J. (2008). Cloning and analyzing of rice blast resistance gene Pi-ta+ allele from Jinghong erect type of common wild rice (*Oryza rufipogon* Griff) in Yunnan. Yi Chuan.

[286] Huang E., Hwang S., Chiang Y., Lin T. (2008). Molecular evolution of the Pi-ta gene resistant to rice blast in wild rice (*Oryza rufipogon*). Genetics.

[287] Wang X., Jia Y., Shu Q.Y., Wu D. (2008). Haplotype diversity at the *Pi-ta* locus in cultivated rice and its wild relatives. Phytopathology.

[288] Lee S., Costanzo S., Jia Y., Olsen K., Caicedo A.L. (2009). Evolutionary dynamics of the genomic region around the blast resistance gene *Pi-ta* in AA genome *Oryza* species. Genetics.

[289] Imam J., Mandal N.P., Variar M., Shukla P. (2016). Allele Mining and Selective Patterns of *Pi9* Gene in a Set of Rice Landraces from India. Front. Plant Sci..

[290] Thakur S., Gupta Y.K., Singh P.K., Rathour R., Variar M., Prashanthi S.K., Singh U.D., Chand D., Rana J.C., Sharma T.R. (2013). Molecular diversity in rice blast resistance gene *Pi-ta* makes it highly effective against dynamic population of *Magnaporthe oryzae*. Funct. Integr. Genom..

[291] Liu J., Hu Y., Ning Y., Jiang N., Wu J., Jeon J.-S., Xiao Y., Liu X., Dai L., Wang G.-L. (2011). Genetic variation and evolution of the *Pi9* blast resistance locus in the AA genome *Oryza* species. J. Plant Biol..

[292] Thakur S., Singh P.K., Rathour R., Variar M., Prashanthi S.K., Singh A.K., Singh U.D., Chand D., Singh N.K., Sharma T.R. (2013). Positive selection pressure on rice blast resistance allele *Piz-t* makes it divergent in Indian land races. J. Plant Interact..

[293] Thakur S., Singh P.K., Das A., Rathour R., Variar M., Prashanthi S.K., Singh A.K., Singh U.D., Chand D., Singh N.K. (2015). Extensive sequence variation in rice blast resistance gene Pi54 makes it broad spectrum in nature. Front. Plant Sci..

[294] Lv Q., Huang Z., Xu X., Tang L., Liu H., Wang C., Zhou Z., Xin Y., Xing J., Peng Z. (2017). Allelic variation of the rice blast resistance gene Pid3 in cultivated rice worldwide. Sci. Rep..

[295] Zhou Y., Lei F., Wang Q., He W., Yuan B., Yuan W. (2020). Identification of Novel Alleles of the Rice Blast-Resistance Gene *Pi9* through Sequence-Based Allele Mining. Rice.

[296] Scardaci S.C., Webster R.K., Greer C.A., Hill J.E., William J.F., Mutters R.G., Brandon D.M., McKenzie K.S., Oster J.J. (1997). Rice blast: A new disease in California. Agron. Fact Sheet Ser..

[297] Puri K.D., Shrestha S.M., Khhatri Chhetri G.B., Joshi K.D. (2009). Leaf and neck blast resistance reaction in Tropical rice lines under greenhouse condition. Euphytica.

[298] Kumar V., Jain P., Venkadesan S., Karkute S., Bhati J., Abdin M., Sevanthi A., Mishra D., Chaturvedi K., Rai A. (2021). Understanding rice-*Magnaporthe oryzae* interaction in resistant and susceptible cultivars of rice under panicle blast infection using a time-course transcriptome analysis. Genes.

[299] Bonman J.M. (1992). Durable resistance to rice blast disease-environmental influences. Breeding for Disease Resistance.

[300] Sirithunya P., Tragoonrung S., Vanavichit A., Pa-In N., Vongsaprom C., Toojinda T. (2002). Quantitative Trait loci associated with leaf and neck blast resistance in recombinant inbred line population of rice (*Oryza sativa*). DNA Res..

[301] Noenplab A., Vanavichit A., Toojinda T., Sirithunya P., Tragoonrung S., Sriprakhon S., Vongsaprom C. (2006). QTL Mapping for leaf and neck blast resistance in Khao Dawk Mali 105 and Jao Hom Nin recombinant inbred lines. Sci. Asia.

[302] Kalia S., Rathour R. (2019). Current status on mapping of genes for resistance to leaf- and neck-blast disease in rice. 3Biotech.

[303] Ishihara T., Hayano-Saito Y., Oide S., Ebana K., La N.T., Hayashi K., Ashizawa T., Suzuki F., Koizumi S. (2014). Quantitative trait locus analysis of resistance to panicle blast in the rice cultivar Miyazakimochi. Rice.

[304] Fang N., Wang R., He W., Yin C., Guan C., Chen H., Huang J., Wang J., Bao Y., Zhang H. (2016). QTL mapping of panicle blast resistance in *japonica* landrace Heikezijing and its application in rice breeding. Mol. Breed..

[305] Biswal A.K., Shamim M., Cruzado K., Soriano G., Ghatak A., Toleco M., Vikram P. (2017). Role of biotechnology in rice production. Rice Production Worldwide.

[306] Ashkani S., Rafii M.Y., Shabanimofrad M., Ghasemzadeh A., Ravanfar S.A., Latif M.A. (2016). Molecular progress on the mapping and cloning of functional genes for blast disease in rice (*Oryza sativa* L.): Current status and future considerations. Crit. Rev. Biotechnol..

[307] Nizolli V.O., Pegoraro C., de Oliveira A.C. (2021). Rice blast: Strategies and challenges for improving genetic resistance. Crop Breed. Appl. Biotechnol..

[308] Wongsaprom C., Sirithunya P., Vanavichit A., Pantuwan G., Jongdee B., Sidhiwong N., Lanceras-Siangliw J., Toojinda T. (2010). Two introgressed quantitative trait loci confer a broad-spectrum resistance to blast disease in the genetic background of the cultivar RD6 a Thai glutinous jasmine rice. Field Crops Res..

[309] Sreewongchai T., Toojinda T., Thanintorn N., Kosawang C., Vanavichit A., Tharreau D., Sirithunya P. (2010). Development of elite indica rice lines with wide spectrum of resistance to Thai blast isolates by pyramiding multiple resistance QTLs. Plant Breed..

[310] Manivong P., Korinsak S., Korinsak S., Siangliw J.L., Vanavichit A., Toojinda T. (2014). Marker-assisted selection to improve submergence tolerance, blast resistance and strong fragrance in glutinous rice. Genom. Genet..

[311] Fukuoka S., Saka N., Mizukami Y., Koga H., Yamanouchi U., Yoshioka Y., Hayashi N., Ebana K., Mizobuchi R., Yano M. (2015). Gene pyramiding enhances durable blast disease resistance in rice. Sci. Rep..

[312] Suwannual T., Chankaew S., Monkham T., Saksirirat W., Sanitchon J. (2017). Pyramiding of four blast resistance QTLs into Thai rice cultivar RD6 through marker-assisted selection. Czech J. Genet. Plant Breed..

[313] Kiyosawa S. (1989). Breakdown of blast resistance in relation to general strategies of resistance gene deployment to prolong effectiveness of resistance in plants. Plant Dis. Epidemiol..

[314] Khanna A., Sharma V., Ellur R.K., Shikari A., Krishnan S.G., Singh U.D., Prakash G., Sharma T.R., Rathour R., Variar M. (2015). Development and evaluation of near-isogenic lines for major blast resistance gene(s) in Basmati rice. Theor. Appl. Genet..

[315] Jiang J., Mou T., Yu H., Zhou F. (2015). Molecular breeding of thermo-sensitive genic male sterile (TGMS) lines of rice for blast resistance using Pi2 gene. Rice.

[316] Tian D., Chen Z., Chen Z., Zhou Y., Wang Z., Wang F., Chen S. (2016). Allele-specific marker-based assessment revealed that the rice blast resistance genes Pi2 and Pi9 have not been widely deployed in Chinese indica rice cultivars. Rice.

[317] Ning X., Yunyu W., Aihong L. (2020). Strategy for use of rice blast resistance genes in rice molecular breeding. Rice Sci..

[318] Xing X., Liu X.L., Chen H.L., Yang F.Y., Li Y.C., Liao H., You L., Liu J.L., Dai L.Y., Wang G.L. (2016). Improving blast resistance of rice restorer R288 by molecular marker-assisted selection of Pi9 Gene. Crop Res..

[319] Huang Y.L., Yan Z., Wang H., Shen G.L., Zhang C.H. (2018). Directed improvement of rice blast resistance of sterile line Q211S with molecular marker-assisted selection. Chin. Agric. Sci. Bull..

[320] Beser N., Del Valle M.M., Kim S.M., Vinarao R.B., Surek H., Jena K.K. (2016). Marker-assisted introgression of a broad-spectrum resistance gene, Pi40 improved blast resistance of two elite rice (*Oryza sativa* L.) cultivars of Turkey. Mol. Plant Breed..

[321] Kumar S.V., Srinivas Prasad M., Rambabu R., Madhavi K.R., Bhaskar B., Abhilash Kumar V., Sundaram R.M., Krishna Satya A., Sheshu Madhav M., Prakasam V. (2019). Marker-assisted introgression of Pi-1 gene conferring resistance to rice blast pathogen pyricularia oryzae in the background of Samba Mahsuri. Int. J. Curr. Microbiol. Appl. Sci..

[322] Kumar S.V., Rambabu R., Bhaskar B., Madhavi K.R., Srikanth S., Prakasam V., Sundaram R.M., Madhav M.S., Rao L.S., Prasad M.S. (2018). Introgression of durable blast resistance gene Pi-54 into indica rice cv. samba mahsuri, through Marker Assisted Backcross Breeding. Electron. J. Plant Breed..

[323] Khan G.H., Shikari A.B., Vaishnavi R., Najeeb S., Padder B.A., Bhat Z.A., Parray G.A., Bhat M.A., Kumar R., Singh N.K. (2018). Marker-assisted introgression of three dominant blast resistance genes into an aromatic rice cultivar Mushk Budji. Sci. Rep..

[324] Arunakumari K., Durgarani C.V., Satturu V., Sarikonda K.R., Chittoor P.D.R., Vutukuri B., Laha G.S., Nelli A.P.K., Gattu S., Jamal M. (2016). Marker-assisted pyramiding of genes conferring resistance against bacterial blight and blast diseases into Indian rice variety MTU1010. Rice Sci..

[325] Xiao W.M., Luo L.X., Hui W.A.N.G., Tao G.U.O., Liu Y.Z., Zhou J.Y., Zhu X.Y., Yang Q.Y., Chen Z.Q. (2016). Pyramiding of Pi46 and Pita to improve blast resistance and to evaluate the resistance effect of the two R genes. J. Integr. Agric..

[326] Liu W., Wang F., Jin S., Zhu X., Li J., Liu Z., Liao Y., Zhu M., Huang H., Fu F. (2008). Improvement of rice blast resistance in TGMS line by pyramiding of Pi1 and Pi2 through molecular marker-assisted selection. Acta Agron. Sin..

[327] Tanweer F.A., Rafii M.Y., Sijam K., Rahim H.A., Ahmed F., Ashkani S., Latif M.A. (2015). Introgression of blast resistance genes (putative Pi-b and Pi-kh) into elite rice cultivar MR219 through marker-assisted selection. Front. Plant Sci..

[328] Yu M., Pan C., Chen X., Yu L., Zhang X., Li Y., Xiao N., Gong H., Sheng S., Pan X. (2013). Resistance spectrum difference between two broad-spectrum blast resistance genes, Pigm and Pi2, and their interaction effect on Pi1. Acta Agron. Sin..

[329] Ellur R.K., Khanna A., Yadav A., Pathania S., Rajashekara H., Singh V.K., Krishnan S.G., Bhowmick P.K., Nagarajan M., Vinod K.K. (2016). Improvement of Basmati rice varieties for resistance to blast and bacterial blight diseases using marker assisted backcross breeding. Plant Sci..

[330] Singh V.K., Singh A., Singh S.P., Ellur R.K., Choudhary V., Sarkel S., Singh D., Krishnan S.G., Nagarajan M., Vinod K.K. (2012). Incorporation of blast resistance into “PRR78”, an elite Basmati rice restorer line, through marker assisted backcross breeding. Field Crops Res..

[331] Xiao N., Wu Y., Pan C., Yu L., Chen Y., Liu G., Li Y., Zhang X., Wang Z., Dai Z. (2017). Improving of rice blast resistances in japonica by pyramiding major R genes. Front. Plant Sci..

[332] Patroti P., Vishalakshi B., Umakanth B., Suresh J., Senguttuvel P., Madhav M.S. (2019). Marker-assisted pyramiding of major blast resistance genes in Swarna-Sub1, an elite rice variety (Oryza sativa L.). Euphytica.

[333] Jiang H., Feng Y., Bao L., Li X., Gao G., Zhang Q., Xiao J., Xu C., He Y. (2012). Improving blast resistance of Jin 23B and its hybrid rice by marker-assisted gene pyramiding. Mol. Breed..

[334] Khanna A., Sharma V., Ellur R.K., Shikari A.B., Krishnan S.G., Singh U.D., Prakash G., Sharma T.R., Rathour R., Variar M. (2015). Marker assisted pyramiding of major blast resistance genes Pi9 and Pita in the genetic background of an elite Basmati rice variety, Pusa Basmati 1. Indian J. Genet..

[335] Usatov A.V., Kostylev P.I., Azarin K.V., Markin N.V., Makarenko M.S., Khachumov V.A., Bibov M.Y. (2016). Introgression of the rice blast resistance genes Pi1, Pi2 and Pi33 into Russian rice varieties by marker-assisted selection. Indian J. Genet. Plant Breed..

[336] Divya B., Robin S., Rabindran R., Senthil S., Raveendran M., Joel A.J. (2014). Marker assisted backcross breeding approach to improve blast resistance in Indian rice (Oryza sativa) variety ADT43. Euphytica.

[337] Ratna Madhavi K., Rambabu R., Abhilash Kumar V., Vijay Kumar S., Aruna J., Ramesh S., Sundaram R.M., Laha G.S., Sheshu Madhav M., Prasad M.S. (2016). Marker assisted introgression of blast (Pi-2 and Pi-54) genes in to the genetic background of elite, bacterial blight resistant indica rice variety, Improved Samba Mahsuri. Euphytica.

[338] Wu Y., Xiao N., Yu L., Pan C., Li Y., Zhang X., Liu G., Dai Z., Pan X., Li A. (2015). Combination patterns of major R genes determine the level of resistance to the M. oryzae in rice (Oryza sativa L.). PLoS ONE.

[339] Jiang H., Li Z., Liu J., Shen Z., Gao G., Zhang Q., He Y. (2019). Development and evaluation of improved lines with broad-spectrum resistance to rice blast using nine resistance genes. Rice.

[340] Balachiranjeevi C.H., Bhaskar N.S., Abhilash V., Akanksha S., Viraktamath B.C., Madhav M.S., Hariprasad A.S., Laha G.S., Prasad M.S., Balachandran S.M. (2015). Marker-assisted introgression of bacterial blight and blast resistance into DRR17B, an elite, fine-grain type maintainer line of rice. Mol. Breed..

[341] Jamaloddin M., Durga Rani C.V., Swathi G., Anuradha C., Vanisri S., Rajan C.P.D., Krishnam Raju S., Bhuvaneshwari V., Jagadeeswar R., Laha G.S. (2020). Marker Assisted Gene Pyramiding (MAGP) for bacterial blight and blast resistance into mega rice variety “Tellahamsa”. PLoS ONE.

[342] Singh V.K., Singh A., Singh S.P., Ellur R.K., Singh D., Gopala Krishnan S., Bhowmick P.K., Nagarajan M., Vinod K.K., Singh U.D. (2013). Marker-assisted simultaneous but stepwise backcross breeding for pyramiding blast resistance genes Piz5 and Pi54 into an elite Basmati rice restorer line ‘PRR 78’. Plant Breed..

[343] Hari Y., Srinivasarao K., Viraktamath B.C., Hari Prasad A.S., Laha G.S., Ahmed M.I., Natarajkumar P., Sujatha K., Srinivas Prasad M., Pandey M. (2013). Marker-assisted introgression of bacterial blight and blast resistance into IR 58025B, an elite maintainer line of rice. Plant Breed..

[344] Singh A., Singh V.K., Singh S.P., Ellur R.K., Singh D., Bhowmick K., Gopala Krishnan S., Nagarajan M., Vinod K.K., Mohapatra T. (2012). Marker aided improvement of Pusa1460 an elite Basmati rice for resistance to Blast diseases. AoB Plants.

[345] Divya B., Biswas A., Robin S., Rabindran R., Joel A. (2014). Gene interactions and genetics of blast resistance and yield attributes in rice (Oryza sativa L.). J. Genet..

[346] Singh J., Dantre R.K., Bhaskar B., Kumar S.V., Madhavi K.R., Prasad M.S. (2018). Performance of Gene Introgressed Lines against Blast Disease under Different Agro Climatic Locations of Chhattisgarh and Telangana. Int. J. Pure Appl. Biosci..

[347] Ravindrababu V., Srinivas Prasad M. (2016). Enhancement of leaf blast resistance in rice cultivar ‘Swarna’ by marker assisted backcross breeding. Int. J. Dev. Res..

[348] Rekha G., Abhilash Kumar V., Viraktamath B.C., Pranathi K., Kousik M.B.V.N., Laxmi Prasanna B., Backiyalakshmi C., Sinha P., Ravindra R.K., Bhaskar S. (2018). Improvement of blast resistance of the popular high-yielding, medium slender-grain type, bacterial blight resistant rice variety, Improved Samba Mahsuri by marker-assisted breeding. J. Plant Biochem. Biotechnol..

[349] Wu Y., Xiao N., Chen Y., Yu L., Pan C., Li Y., Zhang X., Huang N., Ji H., Dai Z. (2019). Comprehensive evaluation of resistance effects of pyramiding lines with different broad-spectrum resistance genes against Magnaporthe oryzae in rice (Oryza sativa L.). Rice.

[350] Ramalingam J., Palanisamy S., Alagarasan G., Renganathan V.G., Ramanathan A., Saraswathi R. (2020). Improvement of stable restorer lines for blast resistance through functional marker in rice (*Oryza sativa* L.). Genes.

[351] Ramkumar G., Madhav M.S., Rama Devi S.J.S., Umakanth B., Pandey M.K., Prasad M.S., Sundaram R.M., Viraktamath B.C., Ravindra Babu V. (2016). Identification and validation of novel alleles of rice blast resistant gene Pi54, and analysis of their nucleotide diversity in landraces and wild Oryza species. Euphytica.

[352] Shikari A.B., Rajashekara H., Khanna A., Krishnan S.G., Rathour R., Singh U.D., Sharma T.R., Prabhu K.V., Singh A.K. (2014). Identification and validation of rice blast resistance genes in Indian rice germplasm. Indian J. Genet. Plant Breed..

[353] Zhang L., Nakagomi Y., Endo T., Teranishi M., Hidema J., Sato S., Higashitani A. (2018). Divergent evolution of rice blast resistance Pi54 locus in the genus Oryza. Rice.

[354] Wan B., Liu K., Zhao S., Zhu J., Liu Y., Zhang G., Zhu G. (2020). Distribution of rice blast resistance genes Pi-ta, Pi-b, Pigm and Pi54 in backbone parent and their relationships with neck blast resistance. Southwest China J. Agric. Sci..

[355] Azizi P., Rafii M.Y., Abdullah S.N., Hanafi M.M., Maziah M., Sahebi M., Ashkani S., Taheri S., Jahromi M.F. (2016). Over-expression of the Pikh gene with a CaMV 35S promoter leads to improved blast disease (Magnaporthe oryzae) tolerance in rice. Front. Plant Sci..

[356] Peng M., Lin X., Xiang X., Ren H., Fan X., Chen K. (2021). Characterization and evaluation of transgenic rice pyramided with the Pi Genes Pib, Pi25 and Pi54. Rice.

[357] Baltes N.J., Voytas D.F. (2015). Enabling plant synthetic biology through genome engineering. Trends Biotechnol..

[358] Thompson D.B., Aboulhouda S., Hysolli E., Smith C.J., Wang S., Castanon O., Church G.M. (2018). The future of multiplexed eukaryotic genome engineering. ACS Chem. Biol..

[359] Devanna B.N., Molla K.A., Parameswaran C., Katara J.L., Kumar A., Panda R.S., Kishor J., Bandita S., Cayalvizhi B., Samantaray S. (2021). CRISPR/Cas mediated genome-editing for rice improvement. ORYZA-Int. J. Rice.

[360] Zaidi S.S., Mukhtar M.S., Mansoor S. (2018). Genome editing: Targeting susceptibility genes for plant disease resistance. Trends Biotechnol..

[361] Müller M., Munné-Bosch S. (2015). Ethylene response factors: A key regulatory hub in hormone and stress signaling. Plant Physiol..

[362] Liu D., Chen X., Liu J., Ye J., Guo Z. (2012). The rice ERF transcription factor OsERF922 negatively regulates resistance to *Magnaporthe oryzae* and salt tolerance. J. Exp. Bot..

[363] Wang F., Wang C., Liu P., Lei C., Hao W., Gao Y. (2016). Enhanced rice blast resistance by CRISPR/Cas9-targeted mutagenesis of the ERF transcription factor gene OsERF922. PLoS ONE.

[364] Ma J., Chen J., Wang M., Ren Y., Wang S., Lei C., Cheng Z. (2018). Disruption of OsSEC3A increases the content of salicylic acid and induces plant defense responses in rice. J. Exp. Bot..

[365] Li S., Shen L., Hu P., Liu Q., Zhu X., Qian Q., Wang K., Wang Y. (2019). Developing disease-resistant thermosensitive male sterile rice by multiplex gene editing. J. Integr. Plant Biol..

[366] Ren B., Yan F., Kuang Y., Li N., Zhang D., Zhou X., Lin H., Zhou H. (2018). Improved base editor for efficiently inducing genetic variations in rice with CRISPR/Cas9-guided hyperactive hAID mutant. Mol. Plant.

